# Prospecting for Novel Plant-Derived Molecules of *Rauvolfia serpentina* as Inhibitors of Aldose Reductase, a Potent Drug Target for Diabetes and Its Complications

**DOI:** 10.1371/journal.pone.0061327

**Published:** 2013-04-17

**Authors:** Shivalika Pathania, Vinay Randhawa, Ganesh Bagler

**Affiliations:** Biotechnology Division, Institute of Himalayan Bioresource Technology, Council of Scientific and Industrial Research, Palampur, Himachal Pradesh, India; University of Akron, United States of America

## Abstract

Aldose Reductase (AR) is implicated in the development of secondary complications of diabetes, providing an interesting target for therapeutic intervention. Extracts of *Rauvolfia serpentina*, a medicinal plant endemic to the Himalayan mountain range, have been known to be effective in alleviating diabetes and its complications. In this study, we aim to prospect for novel plant-derived inhibitors from *R. serpentina* and to understand structural basis of their interactions. An extensive library of *R. serpentina* molecules was compiled and computationally screened for inhibitory action against AR. The stability of complexes, with docked leads, was verified using molecular dynamics simulations. Two structurally distinct plant-derived leads were identified as inhibitors: indobine and indobinine. Further, using these two leads as templates, 16 more leads were identified through ligand-based screening of their structural analogs, from a small molecules database. Thus, we obtained plant-derived indole alkaloids, and their structural analogs, as potential AR inhibitors from a manually curated dataset of *R. serpentina* molecules. Indole alkaloids reported herein, as a novel structural class unreported hitherto, may provide better insights for designing potential AR inhibitors with improved efficacy and fewer side effects.

## Introduction

Diabetes is characterized by irregular carbohydrate metabolism when enough insulin is not produced by pancreas, or when body cannot effectively use the insulin produced, resulting in hyperglycemia. According to the latest World Health Organization estimates, approximately 200 million people all over the world are suffering from diabetes, and this number is expected to cross the 400 million mark by 2030 [1]–[4]. The rise in blood sugar level due to hyperglycaemia is responsible for uncontrolled diabetes, and over the time leads to serious complications affecting renal, cardiovascular, neurological, and optic systems. Diabetes is also known to be a major medical cause of blindness. Approximately half of the diabetic patients die prematurely because of cardiovascular causes, and about 10% from renal failure [2], [3]. Among other pathways studied for their role in diabetes, polyol pathway has been extensively studied and is reported to be central to the mechanisms leading to diabetic complications [5].

**Figure 1:**
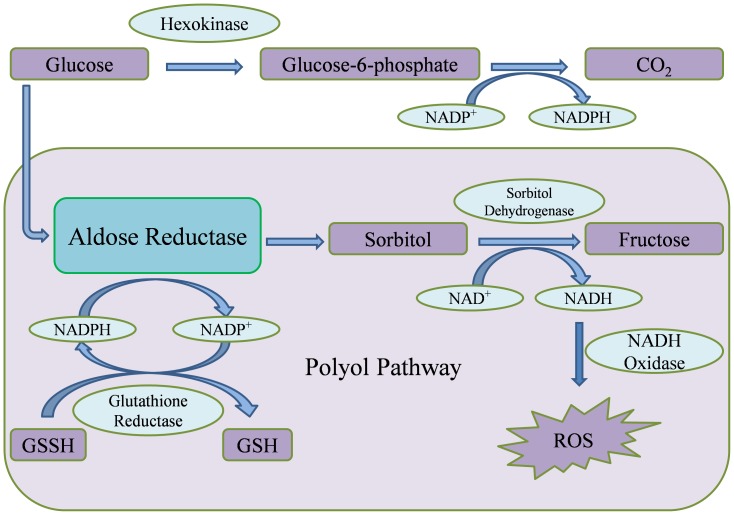
The key role of Aldose Reductase in hyperglycemia-induced oxidative stress. Under normal conditions, glucose is metabolized to release carbon dioxide along with energy. Under hyperglycemic conditions, AR converts glucose to sorbitol, utilizing cofactor NADPH and consequently reduces glutathione level. Further, sorbitol is converted to fructose by NAD^+^ -dependent sorbitol dehydrogenase, leading to production of reactive oxygen species. Intracellular accumulation of sorbitol creates a loss of osmotic integrity and cellular damage, while depletion of NADPH and NAD^+^ cofactors compromises body’s antioxidant defence systems. In addition, high blood levels of fructose may account for increased glycation. These changes result in osmotic and oxidative stresses, ultimately leading to various micro-vascular complications in a number of tissues. Polyol pathway, thus, is involved in various biochemical changes that are relevant to diabetes-induced vascular dysfunction. AR controls the rate-limiting step of polyol pathway, and inhibition of AR provides a possible strategy to prevent complications of chronic diabetes.

Diabetes-induced complications are linked to an enhanced flux of glucose through the polyol pathway. Aldose Reductase (AR, EC 1.1.1.21), an enzyme belonging to aldo-keto reductase superfamily, catalyzes the rate-limiting step of polyol pathway (Figure 1), an alternative path for glucose metabolism [6]. In hyperglycemic conditions, glucose is metabolized through polyol pathway, ultimately leading to production of reactive oxygen species (ROS) [7]. These biochemical changes result in osmotic and oxidative stresses, leading to various micro-vascular complications in a number of tissues, usually aggravating the illness [8]. Polyol pathway is also involved in various biochemical changes such as increased production of advanced glycation end-products and activation of protein kinase C, which could be relevant to diabetes-induced vascular dysfunction [7]. Since AR is a central molecule and is known to control the rate-limiting step of polyol pathway, its inhibition provides a possible strategy to prevent complications of chronic diabetes [9]–[11]. Experimental studies suggest that inhibition of AR could be effective in prevention of diabetic complications [12], [13]. Thus, identifying potent AR inhibitors can pave the way for effective therapies against diabetes and related complications.

Although a large number of Aldose Reductase Inhibitors (ARIs) have been identified, very few of them are known to exhibit sufficient therapeutic efficacy. A number of ARIs, broadly belonging to following three structural classes, have been discovered: acetic acid derivatives, cyclic imides, and phenolic derivatives [14]. Despite numerous efforts made over the last few decades, epalrestat is the only commercially available inhibitor till date [15], [16]. Fidarestat, another drug for diabetic neuropathy [17], has undergone phase III clinical trials and was found to be safe [18]. The failure of new candidate drugs can be assigned to poor pharmacokinetic properties and/or unacceptable side effects [19]–[21]. Hence, there is still a strong need to discover novel ARIs, of diverse structural and chemical features, with potential therapeutic value and lesser side effects. For diabetes and its complications, natural compounds of therapeutic value are highly sought after [22]. Recent studies have reported plant-derived AR inhibitors [23], [24] and data compilations for their exploration [25]. Plant-derived molecules (PDMs) could be effectively used to systematically extract unique molecular scaffolds, which could further be chemically elaborated to generate novel leads and to screen molecules from drug-like libraries [26].

*Rauvolfia serpentina*, commonly known as ‘snakeroot’, is an important medicinal plant endemic to Indian subcontinent and South-East Asian countries [27]. This plant is found in the Himalayan mountain ranges distributed over the foothills up to elevations of 1300–1400 meters. The roots of this plant, known to be having therapeutically important indole alkaloids, are used in the treatment of various diseases [27], [28]. It has also been reported that root extracts from *R. serpentina* exhibit hypoglycemic and hypolipidemic activity against animal models [29], [30]. Based on the reports, we hypothesize that extracts of *R. serpentina* may contain molecules which are active against diabetes and its related complications, potentially through AR inhibition. Hence, towards our aim of identifying natural inhibitors of AR, we planned to compile an extensive library of plant-derived molecules from *R. serpentina* and screen them for inhibitory action.

Computational approaches, such as molecular docking, virtual screening, and molecular dynamics (MD), have been widely used in modern drug discovery to explore drug-receptor interactions [31]–[34]. An efficient way of designing novel inhibitors is to screen molecules from a database of organic compounds based on steric and electrostatic complementarity with the binding pocket of protein [35]. However, the flexibility of protein is not taken into account in docking studies, whereas MD treats both the ligand and protein as flexible entities [36]. Hence, MD simulations can help in further refinement of docked complexes and in obtaining detailed information on structural changes. Therefore, combination of these methods has the potential to reveal mechanisms of drug-receptor interactions, and provide structural insights by which molecules interact within binding pocket of the receptor.

**Figure 2:**
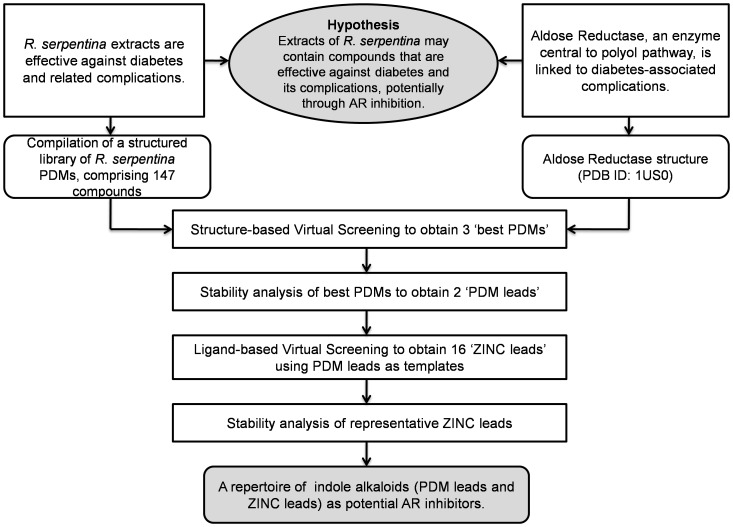
Strategy implemented towards prospecting for novel ARIs from *R. serpentina*. *R. serpentina* extracts are reported to be effective against diabetes and its complications. AR controls the rate-limiting step of polyol pathway, and its inhibition is known to prevent complications of diabetes. Founded in these empirical facts, we propose a hypothesis connecting effectiveness of molecular constituents of plant extracts to a regulatory mechanism central to the disorder. Towards our aim of prospecting for novel ARIs, we compiled a structured library of *R. serpentina* PDMs, and screened them to obtain ‘best PDMs’ (3). The best PDMs were refined to obtain two ‘PDM leads’ on the basis of their structural stability. Further, 16 more ‘ZINC leads’ were identified by screening structural analogs of these plant-derived leads, and representative analogs were assessed for their structural stability. This prospection study presents a repertoire of plant-derived indole alkaloids, and their analogs, as potential AR inhibitors.

In this study, towards our aim of identifying novel ARIs, we first compiled an extensive library of molecules reported from *R. serpentina*. A structured dataset of *R. serpentina* PDMs, inclusive of chemical and structural details, was created. A structure-based molecular docking of these molecules was performed against AR. In order to further refine and examine the stability of three best docked complexes obtained, MD simulations were performed. Two indole alkaloid leads were identified as potential AR inhibitors (‘PDM leads’). Further, to search the neighborhood of chemical space for potential ARIs, structural analogs of the PDM leads were obtained. Starting with these leads as templates, analogs obtained from ZINC [37] database were subjected to virtual screening to identify 16 more potential AR inhibitors (‘ZINC leads’). The strategy implemented in this study is depicted in Figure 2. The leads obtained are promising candidates which could be used for further experimental evaluation and validation.

## Materials and Methods

AR is one of the potent drug targets for diabetes and related complications [10]. *R. serpentina* is reported to contain a large number of alkaloids, a possible source of ARIs. Root extracts from *R. serpentina* are reported to exhibit hypoglycemic and hypolipidemic activity against animal models [29], [30]. This provided us a basis to create library of PDMs and for further computational investigations of their inhibitory action against AR. Towards our goal of identification of potent, novel ARIs from *R. serpentina,* we adopted the following procedure: (1) compilation of an extensive library and structured dataset of PDMs, (2) small-molecule library preparation, (3) receptor preparation, (4) validation of docking protocol, (5) structure-based screening using molecular docking, (6) stability evaluation and refinement of best PDM complexes using MD simulations, (7) ligand-based screening of structural analogs from ZINC database, and (8) stability evaluation of representative ZINC analogs.

### Compilation of Plant-derived Molecules of *R. serpentina*

**Figure 3:**
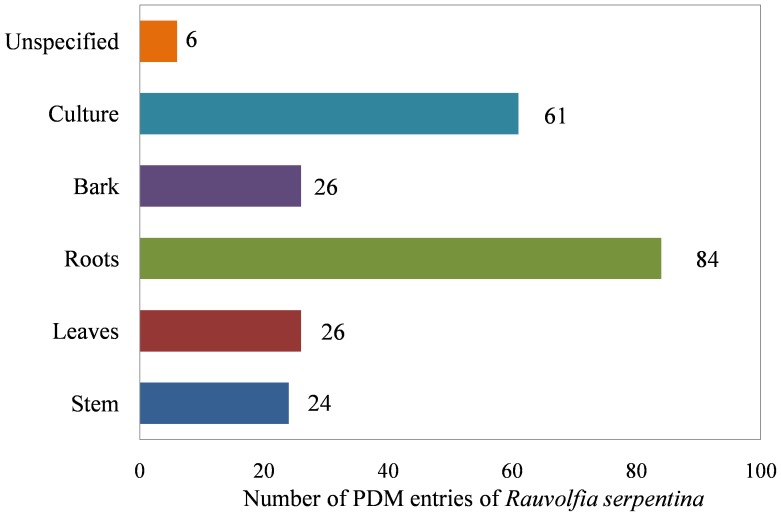
Abundance of entries for *R. serpentina* plant-derived molecules from different plant parts. Number of PDM entries reflecting the abundance of PDMs from different plant parts: stem, leaves, roots, bark, culture, and unspecified. The PDM entry was classified as ‘Unspecified’, when no specific plant part, from which it was extracted, was reported. The plant part class ‘Culture’ includes following sub-categories: hairy root culture, root culture, hybrid cell culture, cell culture, and cell suspension culture.

**Figure 4:**
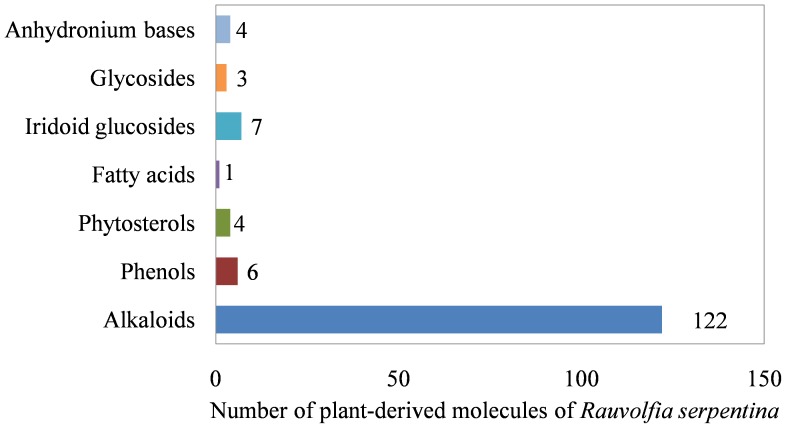
Phytochemical composition of *R. serpentina* plant-derived molecules. Number of PDMs obtained for different structural classes of phytochemicals.

In order to build an extensive library of PDMs from *R. serpentina,* data were compiled from literature and following web resources: A database on antidiabetic plants [38], Global Information Hub On Integrated Medicine [39], and India Herbs [40]. PubMed (http://www.ncbi.nlm.nih.gov/pubmed) was searched with the keywords ‘*Rauvolfia serpentina*’ and ‘*Rauwolfia serpentina*’ to obtain relevant literature. Two variants of spelling were used to address degeneracy in the literature. All the resources were manually curated to extract data of PDMs and their additional details including chemical name, plant part, IUPAC (International Union of Pure and Applied Chemistry) name, and 2D structure. Along with PubMed, above-mentioned web resources were also mined to make the list more extensive and as complete as possible. To authenticate the chemical details obtained, molecules were also ascertained from the Dictionary of Natural Products (DNP) [41]. Additionally, PhD dissertations [42], [43] reporting alkaloid constituents of *R. serpentina*, and following books were used for curation of data: ‘The Alkaloids’ [44], ‘The Alkaloids: Chemistry and Physiology’ [45], and ‘The Alkaloids: Chemistry and Physiology’ [46]. To remove the redundant entries, data from all the resources were merged and an extensive library of PDMs was compiled. The final dataset contained a total of 147 molecules (Table 1 and Table S1) reported to be extracted from various plant parts (Figure 3). The details of their phytochemical composition are provided in Figure 4. A separate entry was created for molecules that were obtained from more than one plant part. The dataset contained 227 such individual entries for 147 molecules. Of these, only 142 molecules were subjected to molecular docking, since chemical structure of 5 molecules could not be obtained. Complete details of the curated and complied PDM dataset are provided in Table S1.

**Table 1:** Dataset of plant-derived molecules of *Rauvolfia serpentina.*

PDM_ID	PDM	PDM_ID	PDM	PDM_ID	PDM
RASE0001	Reserpine	RASE0051	Rauhimbine	RASE0101	Reserpoxidine
RASE0002	Ajmaline	RASE0052	Sandwicolidine	RASE0102	12-Hydroxyajmaline
RASE0003	Isoajmaline	RASE0053	Sandwicoline	RASE0103	3-Epi-alpha-yohimbine
RASE0004	Neoajmaline	RASE0054	Yohambinine	RASE0104	18-beta-hydroxy-3-epi-alpha-yohimbine
RASE0005	Ajmalicine	RASE0055	Sucrose	RASE0105	17-O-acetyl-ajmaline
RASE0006	Raubasine	RASE0056	Oleic acid	RASE0106	Strictosidine
RASE0007	Yohimbine	RASE0057	Fumaric acid	RASE0107	Strictosidine lactam
RASE0008	Deserpidine	RASE0058	γ-Sitosterol	RASE0108	Eburnamonine
RASE0009	Rescinnamine	RASE0059	β-Sitosterol	RASE0109	Stemmadenine
RASE0010	Serpentinine	RASE0060	Serposterol	RASE0110	Akuammigine
RASE0011	Corynanthine	RASE0061	Diisobutyl phthalate	RASE0111	Gardnerine
RASE0012	Papaverine	RASE0062	Deserpidic acid lactone	RASE0112	16-Epigardnerine
RASE0013	Sarpagine	RASE0063	Vallesiachotamine	RASE0113	Isosandwicine
RASE0014	Serpentine	RASE0064	19-Hydroxy- Nβ-methylraumacline	RASE0114	Rauniticine
RASE0015	Serpinine	RASE0065	6α-Hydroxyraumacline	RASE0115	Sandwicine
RASE0016	Alstonine	RASE0066	6α-Methoxyraumacline	RASE0116	Vincoside lactam
RASE0017	Ajmalinine	RASE0067	N(b)-Methylajmaline	RASE0117	Alpha-yohimbine
RASE0018	Chandrine	RASE0068	N(b)-Methylisoajmaline	RASE0118	Beta-yohimbine
RASE0019	Renoxidine	RASE0069	3-Hydroxysarpagine	RASE0119	17,21-O-Diacetylajmaline
RASE0020	Reserpiline	RASE0070	Yohimbinic acid	RASE0120	21-O-Acetylvomilenine
RASE0021	Reserpinine	RASE0071	Isorauhimbinic acid	RASE0121	21-hydroxyraumacline
RASE0022	Ophioxylin	RASE0072	7-Epiloganin	RASE0122	Tubotaiwine
RASE0023	Rauwolscine	RASE0073	6′-O-(3,4,5-trimethoxybenzoyl)glomeratose A	RASE0123	21-Hydroxysarpagan-glucoside
RASE0024	Thebaine	RASE0074	Normacusine B	RASE0124	17-O-Acetylrauglucine
RASE0025	7-Dehydrositosterol	RASE0075	Geissoschizol	RASE0125	17-O-Acetyl-nortetraphyllicine
RASE0026	Stigmasterol	RASE0076	Rhazimanine	RASE0126	17-O-Acetyltetraphyllicine
RASE0027	Starch	RASE0077	18-Hydroxyepialloyohimbine	RASE0127	Suaveoline
RASE0028	2,6-Dimethoxybenzoquinone	RASE0078	Methyl reserpate	RASE0128	Macrophylline
RASE0029	Tetraphyllicine	RASE0079	Loganic acid	RASE0129	Rhazinilam
RASE0030	Raucaffricine	RASE0080	7-Deoxyloganic acid	RASE0130	Acetylcorynanthine
RASE0031	Vomilenine	RASE0081	Secoxyloganin	RASE0131	Raunescine
RASE0032	10-Hydroxy-N(α)-demethyl-19,20-dehydroraumacline	RASE0082	(+)-Isolariciresinol3a-O-beta-D-glucopyranoside	RASE0132	Isosandwicimine
RASE0033	Raumacline	RASE0083	Glomeratose A	RASE0133	Seredine
RASE0034	Raucaffrinoline	RASE0084	16-Epinormacusine B	RASE0134	Ajmalicidine
RASE0035	Perakine	RASE0085	Swertiaside	RASE0135	Ajmalinimine
RASE0036	Vinorine	RASE0086	3,4,5,6-Tetradehydroyohimbine	RASE0136	Raugalline
RASE0037	16-epi-vellosimine	RASE0087	3,4,5,6-Tetradehydro-(Z)-geissoschizol	RASE0137	Perakenine
RASE0038	11-Methoxyvinorine	RASE0088	3,4,5,6-Tetradehydrogeissoschizol	RASE0138	Serpine
RASE0039	Vellosimine	RASE0089	3,4,5,6-Tetradehydrogeissoschizine-17-O-b-d-glucopyranoside	RASE0139	Acetylajmalicidine
RASE0040	1,2-dihydrovomilenine	RASE0090	3-Oxo-rhazinilam	RASE0140	Acetylsandwicoline
RASE0041	17-O-Acetyl-norajmaline	RASE0091	Arbutin	RASE0141	Acetylsandwicolidine
RASE0042	Norajmaline	RASE0092	Ajmalimine	RASE0142	Dehydrogeissoschizine
RASE0043	Rauwolfine	RASE0093	Tryptamine	RASE0143	19(S),20(R)-dihydroperaksine-17,21-al
RASE0044	Rauwolfinine	RASE0094	Secologanin	RASE0144	(+)-17R-O-(3',4',5'-trimethoxybenzoyl)ajmaline
RASE0045	Rescinnamidine	RASE0095	Nβ-Methylraumacline	RASE0145	4,21-secoajmaline
RASE0046	Rescinnaminol	RASE0096	Tetrahydroalstonine	RASE0146	Ajmalicimine
RASE0047	Tetraphylline	RASE0097	19(S),20(R)-dihydroperaksine	RASE0147	Acetylajmalicimine
RASE0048	Indobine	RASE0098	19(S),20(R)-dihydroperaksine-17-al		
RASE0049	Indobinine	RASE0099	10-Hydroxy-19(S),20(R)-dihydroperaksine		
RASE0050	Isorauhimbine	RASE0100	19(S),20(R)-(O)-Acetylpreperakine		

### Small-molecule Library Preparation

A small-molecule library of *R. serpentina* PDMs, including 3D coordinates, was created. The chemical structures of molecules were drawn and edited using MarvinSketch v5.10.0 software (https://www.chemaxon.com), an advanced chemical structure editor. Hydrogens were explicitly added to 2D structures and were saved in 3D MOL2 format. In order to optimize, the molecules were subjected to 500 steps of steepest descent energy minimization with Merck Molecular Force Field (MMFF94) using OpenBabel 2.3.1 software [47]. The energy minimized conformers were used as ligands for molecular docking. Individual MOL2 files were converted into PDBQT format (acceptable format for AutoDock Vina package [48]), using the python script ‘prepare_ligand4.py’ available in Autodock Tools 1.5.4 package [49]. During this conversion, appropriate charges were added to ligands.

### Receptor Preparation

Protein coordinates from the crystal structure of human AR, a monomeric enzyme in complex with a potent inhibitor IDD594 (PDB ID: 1US0) [50], was used for molecular docking studies. This complex was determined at a resolution of 0.66 Å, which is the best resolution structure available for an AR-ligand complex. This structure was selected as the receptor, based on ultrahigh resolution and highest binding affinity of bound inhibitor towards AR, compared to other AR-inhibitor complexes reported [11]. The complex at this resolution provides exact information about the inhibitor and protein conformation. For receptor preparation, all water and solvent molecules present in the PDB file were manually removed prior to docking, as they were not found to play any important role in protein-IDD594 interactions. Using Autodock Tools, polar hydrogen atoms were added and non-polar hydrogen atoms were merged. The protein receptor was converted from PDB to PDBQT format. All other receptor preparation options were kept at default.

### Validation of the Docking Protocol

Before commencing prospective screening for leads, the reliability and robustness of docking protocol to be implemented was validated, using following two methods: (1) Receiver Operating Characteristic (ROC) curve analysis, and (2) comparison of experimental and computationally obtained ligand conformations.

The docking protocol was first investigated for its discriminatory power among actives and decoys by screening them against the AR structure. The Directory of Useful Decoys (DUD) dataset [51] for AR (accessed in October 2012), comprising 26 actives and 995 decoys, was obtained from DUD website (http://dud.docking.org/r2/). The MOL2 formatted files, containing 3D coordinates, were energy minimized with 500 steps of steepest descent using MMFF94 force field. Further, these files were converted to PDBQT format using MGLTools. The actives and decoys were then docked into AR structure using AutoDock Vina 1.1.2 [48] package. The receptor was kept rigid, while the ligands (actives and decoys) were set flexible to rotate and explore most probable binding poses. A rectangular cuboid grid box with dimensions of 25×25×25 points, along the x, y, and z axes, was defined around the binding site to circumscribe it entirely, and to accommodate free motion of ligands. For each run, 100 highest-scoring docking poses were saved and binding affinity of the best mode was selected. As a post-docking filter, those ligands not occupying the binding pocket or not found to be interacting with experimentally observed critical residues (Tyr48, His110, Trp111, and Thr113) [50] were ignored. This procedure reduces the number of false positives. Docking performance was quantified using area under the curve (AUC) by plotting the ROC curve. The ROC was plotted using ROCR package [52] in R-2.15.1statistical package (http://www.r-project.org/).

Docking protocol was also validated by comparing computationally obtained binding conformation of the ligand (IDD594) with that of the experimental conformation observed in the crystal complex (PDB ID: 1US0). Coordinates of bound ligand were extracted from the complex and re-docked into the binding site, using AutoDock Vina, with docking protocol implemented for ROC analysis. Best mode obtained from re-docking procedure was treated as the positive control.

### Molecular Docking and Analysis of Binding Poses

AutoDock Vina was used for all molecular docking simulation studies. The bound inhibitor IDD594 was removed from AR structure, and 142 PDMs of *R. serpentina* were docked into binding site using the validated docking protocol. The docking protocol was implemented 5 times. IDD594 was used to determine search-space size around the binding site. For each of the ligands, 100 feasible binding conformations ranked according to their binding affinities were obtained. At the end of docking run, AutoDock Vina generates docking log files containing records of docking, including binding affinity, for each predicted mode. The program ranks docked conformations based on their binding affinities. Binding affinity represents the sum of total intermolecular energy, total internal energy and torsional free energy minus the energy of unbound system [48]. Molecular interactions between protein and ligands were predicted using Ligplot^+^ v.1.4.3 software [53]. Molecules with binding affinity better than that of the positive control, and those making interactions with critical residues, were selected as ‘best PDMs’. Molecular rendering was performed using PyMOL software (PyMOL Molecular Graphics System, Version 1.5.0.1, Schrödinger, LLC).

### Stability Evaluation by Molecular Dynamics Simulations

In order to refine and examine the stability of docking complexes of all the three best PDMs obtained from molecular docking, MD simulations were performed with GROningen MAchine for Chemical Simulation (GROMACS) 4.0.7 package [54]. GROMACS solves Newtonian equations of motion for the desired system, thereby calculating how atomic coordinates vary as a function of time and tests the stability of complexes. For each complex, independent simulation runs were performed in order to generate trajectories. Before MD simulations, the internal constraints were relaxed by energy minimization.

It is not within the scope of GROMACS to parameterize heteroatom groups in PDB files. Therefore, to include heteroatoms, molecular topology files were generated using Dundee PRODRG server [55]. The complexes were confined into cubic boxes maintaining a minimum of 10 Å between the box edges and the complex surface, while keeping them centered within the box. The resulting systems were then solvated with simple point charge (SPC) 216 water model [56] to yield cuboid boxes of 78×78×78 Å in size. At physiological pH, the structures were found to be positively charged. Therefore, counter ions (2 Cl^−^) were added to neutralize the systems that replaced water molecules at positions of favourable electrostatic potential. Solvated systems were then minimized with 1000 steps of GROMOS96 43a1 force field [57] using steepest descent method, to remove close vander waals contacts. Lennard-Jones interactions were calculated with a cut-off of 1.4 nm, while electrostatic interactions were treated with Particle Mesh Ewald (PME) [58] method using a real space cut-off of 0.9 nm. PME is one of the best methods for computing long-range electrostatics and provides reliable energy estimates. After energy minimization, position restraint dynamics (equilibration run) was performed for 500 picoseconds (ps), where all heavy protein atoms with counter ions were restrained to their starting positions, while allowing water to settle (soak) around the structures. It was performed to avoid unnecessary distortion of structures during simulations. During equilibration of the system, a time step of 2 femtosecond (fs) was used at a temperature of 300 K, and time constant (τT) for temperature coupling was adjusted to 0.1 ps. The box pressure was kept at 1 bar using 1 ps time constant, and a water compressibility of 4.5×10^−5^ bar^−1^ was used. During the run, Linear Constraint Solver (LINCS) algorithm [59] was used to constrain the lengths of hydrogen containing bonds**,** while water molecules were constrained with SETTLE algorithm [60]. The simulations were run under NPT (Number of particles, Pressure and Temperature) conditions, using Berendsen’s coupling algorithm [61] to keep the temperature and pressure constant (P = 1 bar, τP = 0.5 ps; T = 300 K; τT = 0.1 ps). After equilibrating the systems, a 5 nanoseconds (ns) long production simulation (MD run) was conducted with a 2 fs time step at a pressure of 1 bar, and a temperature of 300 K, to confirm stability of the systems.

To ensure that docking complexes were well equilibrated, before data were used for further analysis, trajectories in aqueous solution were analyzed. The analyses included plotting of potential energy, root mean square deviations (RMSD), root mean square fluctuations (RMSF), and intermolecular hydrogen bonds, using g_energy, g_rms, g_rmsf, and g_hbond modules, respectively. The trajectories of simulations were plotted using Gnuplot 4.6.0 program (http://www.gnuplot.info).

### Ligand-based Screening of Structural Analogs and Stability Analysis

After identifying two PDM leads from MD simulations, they were used as a reference to find similar molecules in ZINC database [37]. ZINC is a public database containing over 20 million commercially available compounds in biologically relevant representations. It was hypothesized that the chemical space vicinity of leads may contain molecules with similar chemical properties [62]. The PDM leads were used as structural templates to perform identity-based filtering of ZINC, and their structural analogs with more than 90% identity were obtained. The hits obtained from ZINC were energy minimized using 500 steps of steepest descent with MMFF94 force field, and were further subjected to screening based on molecular docking studies. The binding affinities and modes of hits were investigated using AutoDock Vina.

Two representative molecules were selected, one each from 2 sets of ZINC leads, based on binding affinity and consistency in interactions with critical residues in all the docking runs. In order to validate the stability of complexes of these 2 representative molecules, they were further subjected to energy minimization, position restrained simulations, and MD simulations, using the protocol established earlier.

### Computational Assessment of the Leads Obtained

To ascertain the reliability of PDM leads as well as that of ZINC leads obtained, ROC curve for AR DUD actives and AR DUD decoys was recomputed by appending them with the leads, and their corresponding decoys. DecoyFinder [63] searches for molecules which are physically similar yet chemically different from active ligands. DecoyFinder v1.1 was used at default settings to retrieve decoys for PDM leads, ZINC leads, as well as for AR DUD actives, from the “drug-like” subset of ZINC [64] (accessed in February 2012), which is tailored to extended Lipinski’s rule of five. The subset contained approximately 14,322,885 drug-like molecules.

All computations were performed on an HPZ600 workstation and HP ProLiant DL980 G7 server running Ubuntu 11.10 and Red Hat 4.1.2 operating systems, respectively, with Intel Xeon processors.

## Results

### Compilation of Plant-derived Molecules of *R. serpentina*

While molecules of *R. serpentina*, having potential therapeutic value, are reported in the literature, a comprehensive and structured compilation is hitherto not available. Towards our goal of screening *R. serpentina* molecules, we created an extensive compilation of 147 PDMs reported in the literature, inclusive of following details: chemical name, plant part, IUPAC name, and 2D structure (Table 1 and Table S1). A total of 84 out of 227 PDM entries (37%) were reported from roots, whereas 61 (27%) were reported from cell cultures (Figure 3). Rest of the PDM entries were evenly distributed among molecules reported from bark (26), leaves (26), and stem (24). The PDMs predominantly constituted of indole alkaloids (83%), as observed from their phytochemical composition (Figure 4). A computational pipeline for structure-based virtual screening of molecules from this dataset was implemented. We could not obtain chemical structures of 5 PDMs (PDM_IDs: RASE0017, RASE0018, RASE0027, RASE0032, and RASE0082); hence, these were not considered for screening. Thus, molecular docking studies were performed for 142 PDMs.

### Accuracy of Docking Protocol

The accuracy of docking protocol was evaluated in terms of AUC obtained from the ROC curve analysis. After performing molecular docking, using AutoDock Vina [48], molecules in the DUD dataset were ranked based on their predicted binding affinities. These energy rankings, along with the criterion of interaction with critical residues, were used to evaluate the ability of protocol to preferentially select the actives from decoys. Application of this procedure has been reported to improve the performance of ROC analysis [65].

**Figure 5:**
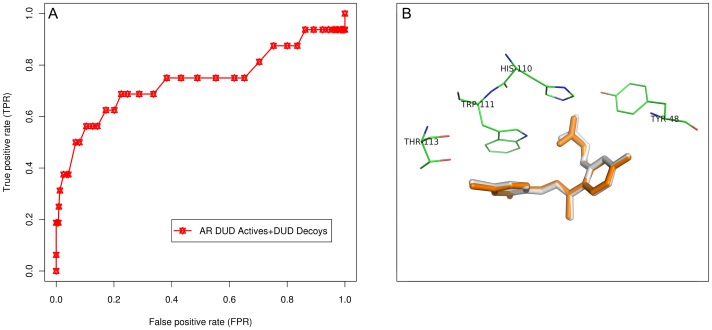
Validation of the docking protocol. (A) ROC curve against AR DUD dataset. ROC statistics shows the success of docking protocol implemented in discriminating actives from decoys. AUC of 0.74 was obtained on the basis of binding affinity scores and interactions with critical residues. ROC curve depicts the true positive rate (sensitivity) versus false positive rate (1-specificity). The graph was rendered using ROCR package. (B) Comparison of experimental and computationally predicted docked conformations of the ligand. Overlay of the experimental (orange) and predicted docked conformation (gray) of IDD594 ligand in the binding site of the receptor (AR; PDB ID: 1US0) with RMSD of 0.094 Å. The figure was rendered using PyMol software.

The use of ROC curve has been observed to be advantageous over other conventional enrichment curves, since they are independent of proportion of actives in the test set, and as they include information on sensitivity as well as specificity [66]. In general, given a set of known actives and decoys, number of actives found among the top-*n* ranked ligands is plotted against *n*, where *n* is size of the dataset. The curve generated is known as ROC, and AUC is given as a fraction of the total plot area. The ROC scores vary between 0 and 1.0; closer the score to 1.0, better is the classifier at distinguishing actives from decoys. The ROC curve analysis for docking results yielded an AUC of 0.74 (Figure 5A), which means that a randomly-selected active had a higher score than a randomly selected decoy 7.4 times out of 10. Since the AUC value was beyond 0.50, docking screening performed better than random discrimination of actives and decoys. This value indicated that the protocol implemented was able to differentiate actives, from decoys, accurately. Sixteen DUD actives retrieved from a total of 26 also corroborated the ROC statistics.

During computational prediction of ligand-binding poses, accuracy of a molecular docking program is usually measured by the RMSD between experimentally observed heavy atom positions of the ligand, and those predicted by the program (usually 1.5–2 Å) [67]. The docking protocol was validated by reproducing experimental binding pose of IDD594 ligand in the binding site of AR. The lowest energy conformation generated during the docking runs was considered as the positive control. The heavy atom RMSD between IDD594 pose and positive control was observed to be 0.094 Å, confirming the quality of docking protocol used, and its suitability for predicting reliable binding modes of leads. Figure 5B shows experimental and docked conformations inlaid into the binding site. Additionally, critical interactions observed in experimental inhibitor-receptor complex were also reproduced in the best computational conformation. Binding affinity of the positive control (−10.7 kcal/mol) was used as a threshold to screen PDM poses obtained from docking studies. These results confirmed the accuracy of docking protocol implemented.

### Structure-based Screening Using Molecular Docking

Molecular docking has been proven to be a reliable means of screening inhibitors from molecular libraries [33], [68], [69]. Docking studies were carried out with *R. serpentina* PDMs in order to find their optimal conformations in the binding pocket of AR. For each of the 142 molecules, the validated docking protocol was implemented 5 times. Nine molecules were initially screened based on their binding affinity values, such that their binding affinity was equal to or better than that of the positive control in at least one of the docking runs (Table S2). Six of these PDMs consistently returned better binding affinities in all the 5 runs: RASE0048, RASE0049, RASE0125, RASE0126, RASE0142, and RASE0143. Rest of the PDMs (RASE0007, RASE0070, and RASE0071) presented poses of desired binding affinities in 3, 2, and 1 docking run(s), respectively.

**Figure 6:**
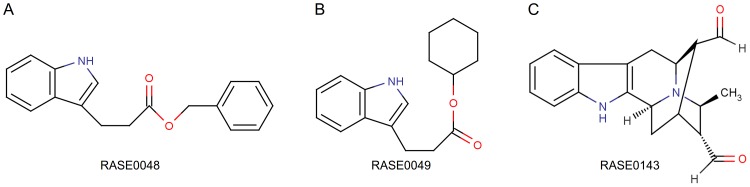
Three best PDMs identified using molecular docking. 2D structures of 3 best PDMs of *R. serpentina* identified on the basis of binding affinity and interactions with critical residues: (A) RASE0048, (B) RASE0049, and (C) RASE0143.

**Figure 7:**
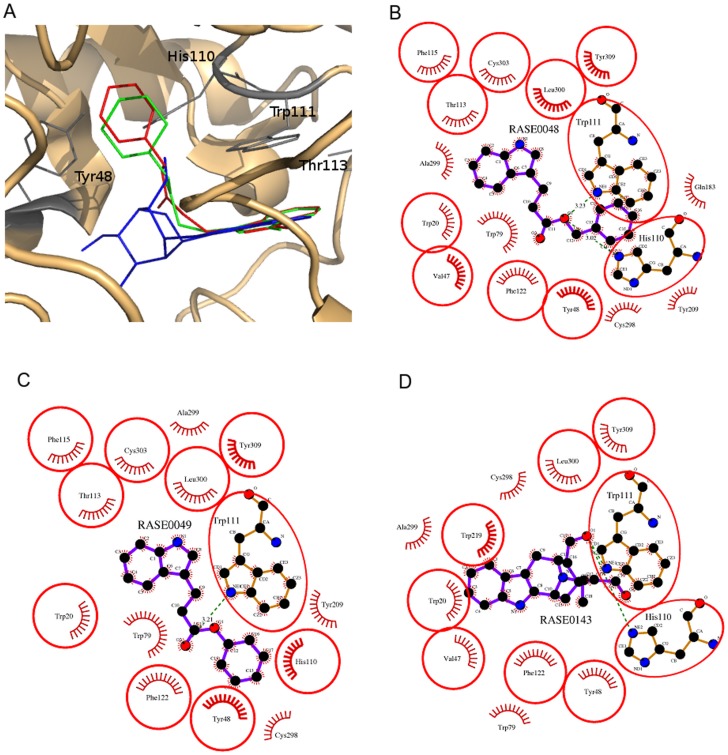
Details of inhibitory interactions made by best PDMs. (A) Three best PDMs, RASE0048 (red), RASE0049 (green), and RASE0143 (blue), docked in the binding site of AR, were visualized as cartoons displaying the catalytic center. 2D interaction plots of docked molecules into the binding site: (B) RASE0048, (C) RASE0049, and (D) RASE0143. Dotted green lines represent hydrogen bonds with constraints, while red spoked arcs represent residues making hydrophobic contacts with ligand. Red circles and ellipses indicate protein residues that are in equivalent 3D positions.

These 6 molecules were further screened on the basis of their chemical interactions with critical residues at the binding site. The residues Tyr48, His110, Trp111, and Thr113 are reported to be involved in making critical inhibitory protein-ligand interactions in the AR-IDD594 complex [14], [50], and were considered as critical for inhibitory mechanisms. A similar conclusion about the nature of the binding site residues could be drawn from structure of AR complexed with inhibitor 4-(20,40-dinitro-anilino)phenol [70]. Finally, 3 best PDMs (RASE0048, RASE0049, and RASE0143) were identified for AR (Figure 6 and Table 2). Each of these 3 molecules, in their best binding poses, was observed to be making interactions with residues known to be crucial for AR inhibition (Figure 7 and Table 2). RASE0048 (indobine) and RASE0143 (19(S),20(R)-dihydroperaksine-17,21-al) were found to be forming hydrogen bonds with Trp111 and His110, whereas RASE0049 (indobinine) was found to be making a hydrogen bond with Trp111. The hydrogen bonding pattern in few of the residues was very similar to that in the AR-IDD594 complex. Hydrogen bonds were made by ‘an oxygen atom (O1) of indobine’ with the ‘nitrogen atom (NE1) of indole group in Trp111’ and another with ‘a nitrogen atom (NE2) of imidazole group in His110’. Identical interactions were observed in RASE0143, whereas in case of RASE0049 ‘a carbon atom (C1) of indobinine’ formed hydrogen bond with ‘nitrogen atom (NE1) of indole group in Trp111’. In addition to hydrogen bonds, best PDMs were also found to be involved in making hydrophobic interactions with receptor, thus stabilizing the complex further (Figure 7). The nature of chemical interactions of these 3 best PDMs, with residues critical for inhibitory interactions. reflect their potential value for therapeutic intervention. For further validation of stability of complexes, MD simulations were carried out.

**Table 2:** Details of binding affinity and hydrogen bond interactions for three ‘leads’ obtained from *R. serpentina* PDMs.

	Binding Affinity (kcal/mol) [number of modes]				
PDM_ID	Run 1	Run 2	Run 3	Run 4	Run 5
RASE0048	−11.0[12]	−11.0[20]	−11.0[20]	−11.1[19]	−11.0[14]
RASE0049	−11.1[14]	−11.0[18]	−11.0[10]	−11.0[12]	−11.0[14]
RASE0143	−10.7[03]	−10.7[03]	−10.7[04]	−10.7[02]	−10.7[04]
	**Hydrogen Bond Interactions [bond length, Å]**				
**PDM_ID**	**Run 1**	**Run 2**	**Run 3**	**Run 4**	**Run 5**
RASE0048	Trp111[3.27]	Trp111[3.23]	Trp111[3.07]	Trp111[3.14]	Trp111[3.08]
RASE0049	His110[2.94]	His110[3.02]	His110[3.08]	His110[3.11]	His110[3.00]
	Trp111[3.20]	Trp111[3.21]	Trp111[3.12]	Trp111[3.12]	Trp111[3.09]
RASE0143	Trp111[3.25]	Trp111[3.25]	Trp111[3.26]	Trp111[3.26]	Trp111[3.25]
	His110[3.23]	His110[3.22]	His110[3.24]	His110[3.23]	His110[3.22]

### Stability Evaluation of the Docked Complexes

MD simulations present an approach for structural refinement of docked complexes. In order to refine and examine the stability of best PDM complexes, MD simulations lasting 5ns were performed and data were collected for further analysis. The systems studied had approximately 47642, 47639, and 47645 atoms for RASE0048, RASE0049, and RASE0143 in complex with AR, respectively, inclusive of 3154 atoms of the protein. The trajectories were analyzed for potential energy and RMSD of Cα backbone atoms with respect to the starting conformation, as a function of time. The potential energy of a system is considered to be a simple measure of its stability. Analyses of trajectories revealed that potential energy of docking complexes decreased gradually in the beginning, with respect to initial energy, and then fluctuated around a flat basal line (Figure S1). This indicated that the systems acquired energetically stable states. The potential energies of AR-indobine and AR-indobinine docking complexes were almost similar; however, it was slightly less for AR-19(S),20(R)-dihydroperaksine-17,21-al complex. The energy values for all 3 complexes were within a narrow range of −6.54e+05 and −6.57e+05 kJ/mol, throughout the dynamics.

**Figure 8:**
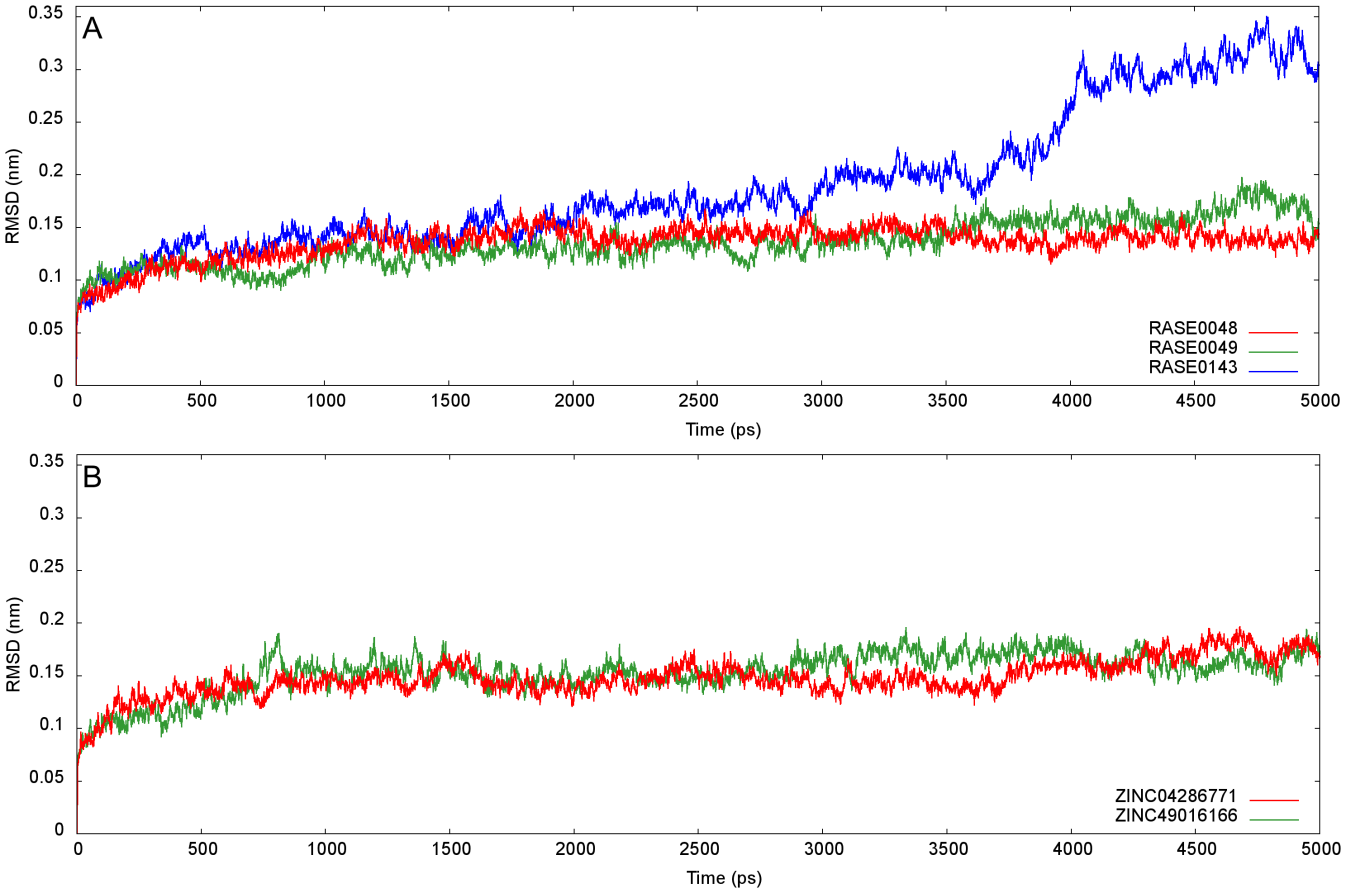
Stability evaluation of docked complexes using RMSD. RMSD profiles of Cα backbone atoms with respect to the starting conformation, as a function of time: (A) Best PDMs and (B) Representative molecules from analogs of PDM leads.

Convergence of structure, measured in terms of RMSD of Cα backbone atoms from the initial structure, is a major criterion used to evaluate the stability. RMSD were observed to increase in first few picoseconds of simulations, thereby indicating substantial conformational changes in protein backbones (Figure 8A). These changes reflect optimization of interactions within the protein structure, as well as with water molecules. The docking complexes of RASE0048 and RASE0049 plateaued to an average RMSD of around 0.14 and 0.16 nm after ∼1.2 and ∼3.6 ns, respectively. RMSD values converged and remained stable till the end of simulations, indicating stable conformations for both the complexes. On the contrary, RMSD of RASE0143 complex was less pronounced and increased gradually during the course of simulation, indicating an unstable system.

**Figure 9:**
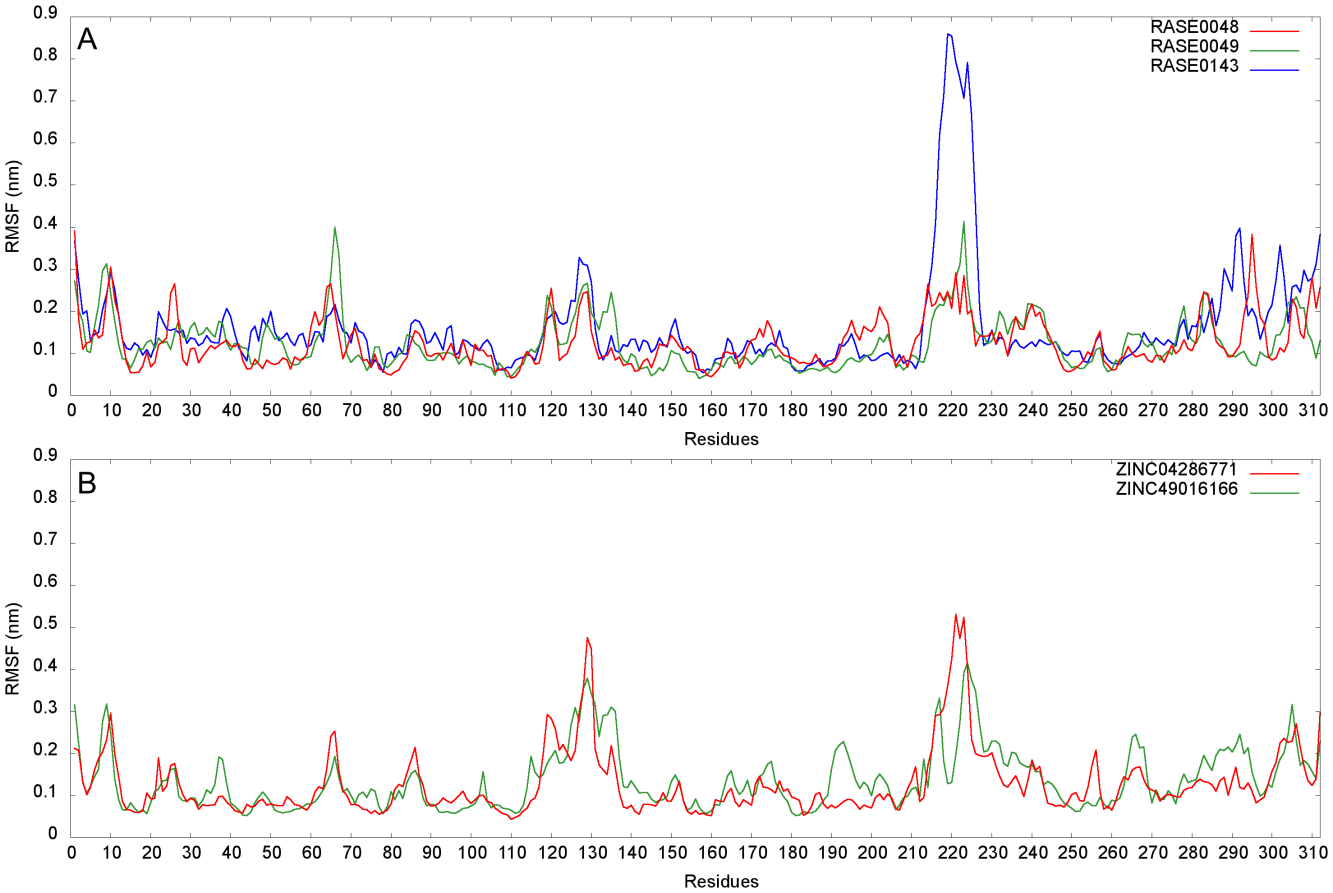
Stability evaluation of docked complexes using RMSF. Local conformational changes in structure as indicated by RMSF of individual residues: (A) Best PDMs and (B) A representative molecule from analogs of PDM leads.

**Figure 10:**
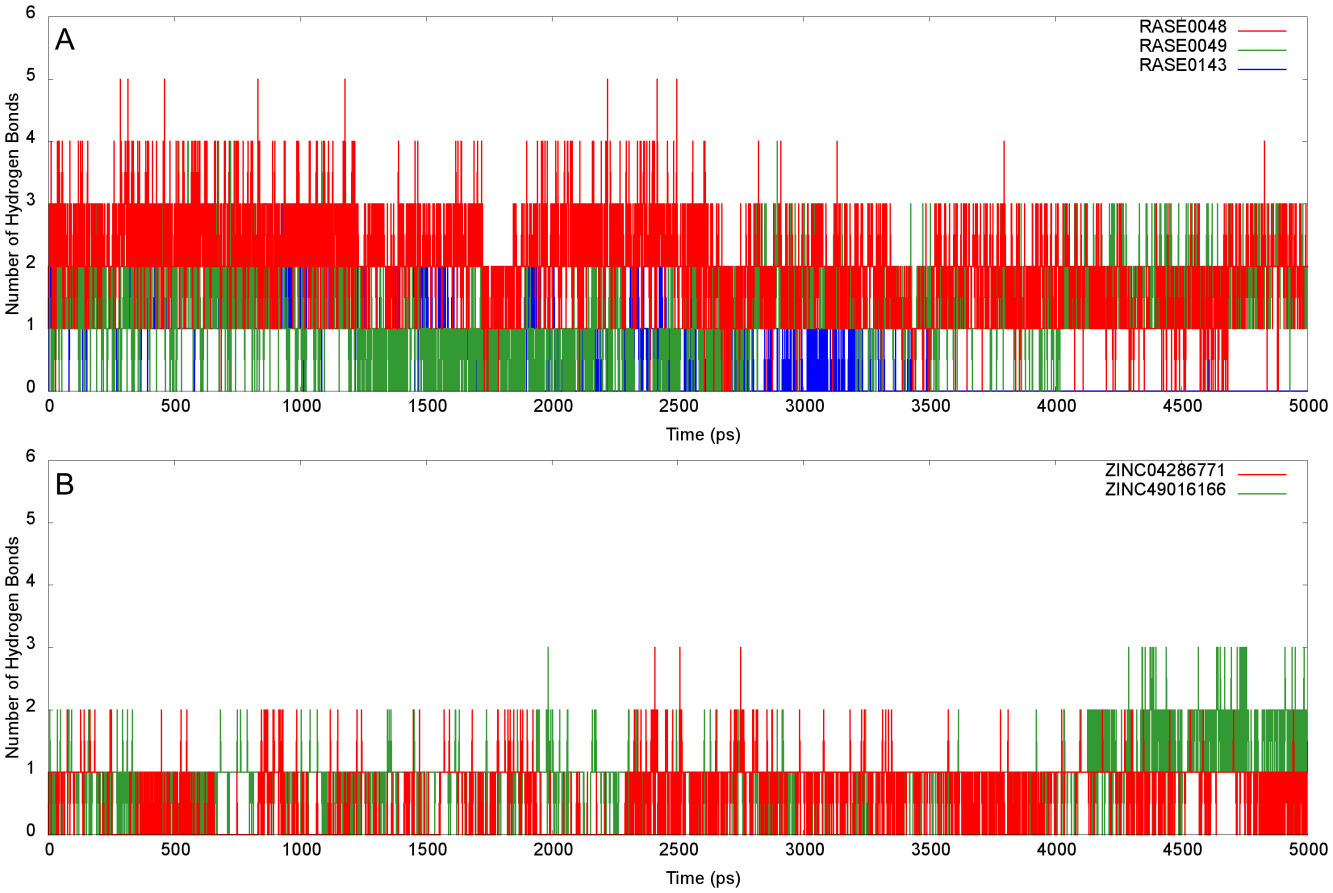
Stability evaluation of docked complexes using hydrogen bonding pattern. Time series plot of number of intermolecular hydrogen bonds: (A) Best PDMs and (B) A representative molecule from analogs of PDM leads.

While RMSD is a measure of global backbone deviation, RMSF captures local changes in the structure. Based on the flexibility of individual residues, relative structural fluctuations of complexes were characterized by plotting RMSF of backbone atoms. None of the critical residues (Tyr48, His110, Trp111, and Thr113) present at the binding site showed large flexibility, indicating that these were not disturbed during ligand binding (Figure 9A). RMSFs in loop regions were comparatively higher than in structured regions, as they are characterized by inherent structural flexibility, compared to secondary structure elements. In case of RASE0143, overall flexibility of the protein structure increased upon ligand binding. Complexes of RASE0048 and RASE0049 had tolerable fluctuations in the backbone, confirming their stability. Further, stability of complexes was confirmed by analyzing time series plots of hydrogen bonds. Figure 10A depicts number of intermolecular hydrogen bonds during simulations. Hydrogen bonds made by RASE0048 with AR remained stable, whereas in case of RASE0049, the bonds broke and reformed quite frequently. The latter formed an extra hydrogen bond, in addition to the bond observed after molecular docking, in the last few nanoseconds. RASE0143 showed a poor hydrogen bonding pattern throughout the simulation, and did not present any hydrogen bond towards the end. The g_hbond module computes number of intermolecular hydrogen bonds, and does not provide information of specific residues involved. Hence, to probe the role of critical residues in making hydrogen bonds, each complex was analyzed at intervals of 0.5 ns using Ligplot^+^ (Table S3). It was observed that RASE0048 and RASE0049 consistently made interactions with critical residues, further confirming and complementing results obtained from RMSD, RMSF, and hydrogen bond time series analysis.

**Figure 11:**
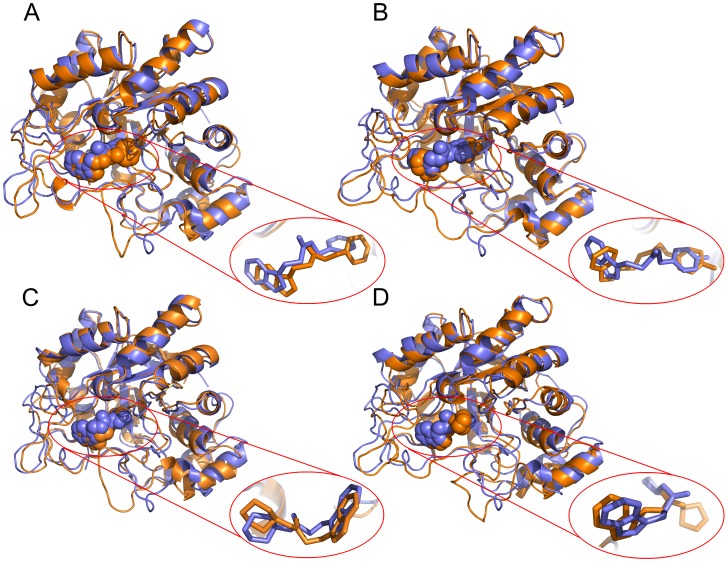
Conformations of PDM leads and ZINC leads, before and after MD simulations. Comparison of conformations of lead complexes before (blue) and after (orange) MD simulations. PDM leads: (A) RASE0048 and (C) RASE0049; ZINC leads: (B) ZINC04286771 and (D) ZINC49016166. AR-lead complexes were superimposed based on the Cα backbone atoms in the average structure obtained, over the initial docked structure.

Based on the above observations from MD studies, 3 best PDMs were refined to 2 ‘PDM leads’: indobine (RASE0048) and indobinine (RASE0049). An average representative structure was computed for both the complexes, from the plateau region. Since the average structures tend to be crude; they were further refined by steepest descent energy minimization. Superimposition of initial docked structures and their average structures are shown in Figure 11A (RASE0048) and Figure 11C (RASE0049). No significant differences were observed between average structures and docked models of the complexes. The protein fold was not disturbed to a large extent, and PDM leads were also presented in almost similar orientations as observed after docking. We conclude that both the PDM lead complexes remained energetically and structurally stable after acquiring the equilibrium state, proving the reliability of docking complexes.

### Screening and Stability Analysis of Structural Analogs

Based on MD studies, each of the 2 PDM leads was used as a template for screening molecules from ZINC database [37]. To search the neighborhood of chemical space for potential ARIs and to understand chemical basis of inhibitory interactions, structural analogs of leads were screened. Molecules in the same neighborhood of chemical space tend to have similar values of a property [71]. We hypothesized that searching the neighborhood of chemical space may yield more potential inhibitors of AR. ZINC molecules with more than 90% identity for each of the 2 PDM leads were identified. A total of 10 ZINC analogs were obtained for RASE0048, whereas 12 were obtained for RASE0049. Energy minimized conformers of these analogs were subjected to molecular docking with AR, implementing the docking protocol established earlier. The analogs obtained were further screened on the basis of their binding affinities and chemical interactions with AR (Table S4 and Table S5). It was found that all 10 analogs of indobine (RASE0048), in their best poses, had binding affinities better than that of the positive control and were observed to be making key inhibitory interactions (Table S4). This supports the chemical basis for mode of action of AR binding, and inhibitory nature of indobine as a novel plant-derived AR inhibitor. From the analogs of RASE0049, 6 ARIs were obtained (Table S5). Thus, overall 16 more indole alkaloids were obtained as potential ARIs (Table S6).

Two representative molecules were selected, one each from 2 sets of ZINC leads, based on binding affinity and consistency in interactions with critical residues in all the docking runs. ZINC04286771 and ZINC49016166 were considered as representatives among analogs of RASE0048 and RASE0049, respectively. Stability of complexes of these molecules was assessed with MD analysis, using the protocol implemented earlier. Based on potential energies (Figure S1), RMSD (Figure 8B), RMSF (Figure 9B), and hydrogen bond time series analysis (Figure 10B), it was concluded that these complexes acquired a state of energetic and structural stability. The intermolecular hydrogen bonding pattern between leads and critical residues is depicted in Table S7. Superimposition of initial docked structures and their corresponding average structures, computed from MD studies, are depicted in Figure 11B (ZINC04286771) and Figure 11D (ZINC49016166).

### Reliability and Novelty of Leads Obtained

**Figure 12:**
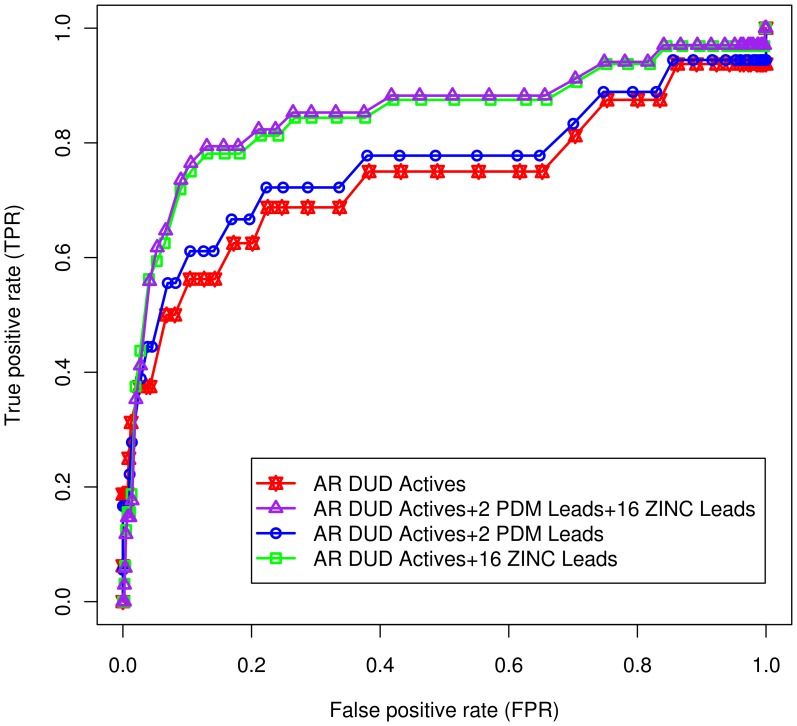
ROC curve analysis for lead sets. To assess the reliability of PDM leads and ZINC leads, ROC curve analysis was performed. For AR DUD actives, corresponding DUD decoys were used (stars; AUC: 0.74). For lead sets, decoys were obtained through DecoyFinder. The DUD actives and decoys were appended with lead sets and their corresponding DecoyFinder decoys, independently (circles and squares) as well as together (triangles). When appended with both the lead sets, AUC improved to 0.85; whereas it improved to 0.76 and 0.84, when appended with PDM leads and ZINC leads, independently.

Reliability of PDM leads (2) as well as that of ZINC leads (16) was ascertained using ROC curve analysis. Decoy sets, corresponding to lead sets, were obtained at default settings of DecoyFinder to yield 36 decoys for each lead. The AR DUD actives (26) and their decoys (995) were appended with leads obtained (2+16) and their DecoyFinder decoys (72+576), respectively. ROC curve analysis was performed for the lead sets, together as well as independently, and compared to that of the AR DUD set (Figure 12). It was observed that the AUC for lead sets improved (0.85) from that of the AR DUD set (0.74), indicating that discrimination ability of docking protocol improved in the presence of lead sets. This highlights potential active-like properties of indole alkaloids obtained as leads in this study. The AUC for PDM leads and ZINC leads (appended to AR DUD set) were 0.76 and 0.84, respectively, further confirming the observation. To check the reliability of decoys obtained through DecoyFinder, ROC curve analysis of AR DUD actives and their DecoyFinder decoys was performed against AR (Figure S2). Further, the novelty of lead sets was confirmed using PubChem (http://pubchem.ncbi.nlm.nih.gov), DrugBank (http://www.drugbank.ca) and ChEMBL (https://www.ebi.ac.uk/chembl) chemical structure databases.

## Discussion

Diabetes-induced complications are known to be associated with enhanced flux of glucose through polyol pathway. AR catalyzes the rate-limiting step of polyol pathway (Figure 1) and provides a potent therapeutic drug target for diabetes and its complications. AR has been extensively investigated, using experimental as well as computational approaches, to develop novel and structurally diverse potent inhibitors [14]–[19], [21], [23]–[25]. *R. serpentina* has been used in Ayurvedic medicinal preparations for thousands of years, and also in tribal ethnomedical systems [27]. Extracts of this plant, reported to have therapeutically important indole alkaloids, show hypoglycaemic and hypolipidemic activity against animal models [29], [30]. We hypothesized that extracts of *R. serpentina* may contain molecules which are active against diabetes and its related complications, potentially through AR inhibition.

To test our hypothesis, we implemented a composite strategy involving data compilation, molecular docking, and MD simulations (Figure 2). We compiled an extensive library of *R. serpentina* molecules. While our compilation of PDMs is extensive, it is neither comprehensive nor complete. There is a lot of scope to enhance the dataset by including molecules hitherto unknown. Using molecular docking approach, we identified 3 structurally distinct PDMs as best candidates (Figure 6): indobine, indobinine, and 19(S),20(R)-dihydroperaksine-17,21-al. The energetic and structural stability of complexes obtained with the best PDMs were assessed using molecular dynamics simulations, to obtain 2 PDM leads. Further, 16 more novel ZINC leads were obtained from structural analogs of PDM leads by spanning their chemical space.

Docking protocol implemented in this work has been thoroughly verified using the ROC statistics (Figure 5A), and by comparing the experimental conformation of bound inhibitor (IDD594) with that obtained from docking studies (Figure 5B). We also checked the reliability of our protocol by performing docking of AR with 4,5-Di-*O*-caffeoylquinic acid, a compound reported to be an effective AR inhibitor from *in vitro* studies. It has been reported that this compound, extracted from *Artemisia dracunculus*, reduced AR activity by 77% at 3.75 µg/mL [72]. Interestingly, using our docking protocol, we found that 4,5-Di-*O*-caffeoylquinic acid emerged as a potent AR inhibitor with a very strong binding affinity and made inhibitory interactions with critical residues. This further lends a strong support for computational procedures used in our work.

Based on the observations from molecular docking and stability analysis, we conclude that indobine (RASE0048) is the most potent ARI candidate. Among the 3 best PDMs obtained, we observed that the chemical interactions established between indobine and AR binding pocket residues were the most stable. All 10 structural analogs of indobine qualified as potential leads, further strengthening the case for mode of action of its inhibitory interactions (Table S4). These observations also support possible search for potent ARIs starting with indobine’s scaffold in order to span the neighborhood of its chemical space. The pattern of molecular interactions observed in these studies may enable efforts to design novel structures with potential inhibitory action.

Earlier studies have implemented similar computational strategies to identify ARIs [73]–[75]. In the light of mechanisms known to be central to diabetes and its complications, we proposed that PDMs of *R. serpentina* could be prospected for, in search of potential aldose reductase inhibitors. We believe that leads identified in this study could provide insight for designing novel inhibitors of aldose reductase with better efficacy and fewer side effects. To the best of our knowledge, this is the first report of screening ARIs from *R. serpentina* for treating diabetes and related complications. While so far ARIs belonging to three major classes, namely acetic acid derivatives, cyclic imides, and phenolic derivaties, have been reported [14], herein we report 2 plant-derived indole alkaloids and their 16 structural analogs as potential ARIs.

## Supporting Information

Figure S1 **Potential energy profiles of best PDMs and representative ZINC leads.** Potential energies of complexes, as a function of time: (A) Best PDMs and (B) Representative molecules from analogs of PDM leads.
(TIFF)

Figure S2 **ROC analysis to evaluate reliability of DecoyFinder decoys.** Comparison of results from ROC curve analysis for the AR DUD actives against (A) AR DUD decoys and (B) AR DecoyFinder decoys. The AUC for the former was 0.74, whereas that for the latter was 0.64, asserting the reliability of DecoyFinder.
(TIFF)

Table S1 **A structured dataset of** ***R. serpentina*** **PDMs.**
(PDF)

Table S2 **Nine** ***R. serpentina*** **PDMs with desired binding affinities.**
(PDF)

Table S3 **Hydrogen bonding pattern with critical residues (for best PDMs).**
(PDF)

Table S4 **Binding affinities and hydrogen bond interactions for analogs of RASE0048.**
(PDF)

Table S5 **Binding affinities and hydrogen bond interactions for analogs of RASE0049.**
(PDF)

Table S6 **Sixteen indole alkaloids obtained by screening the structural analogs of two PDM leads.**
(PDF)

Table S7 **Hydrogen bonding pattern with critical residues (for representative ZINC leads).**
(PDF)
