# Supplementary figures and images for: Prospecting for Novel Plant-Derived Molecules of Rauvolfia serpentina as Inhibitors of Aldose Reductase, a Potent Drug Target for Diabetes and Its Complications
Source: PLoS One. 2013 Apr 17;8(4):e61327. doi: 10.1371/journal.pone.0061327 (PMC3629236; doi:10.1371/journal.pone.0061327)

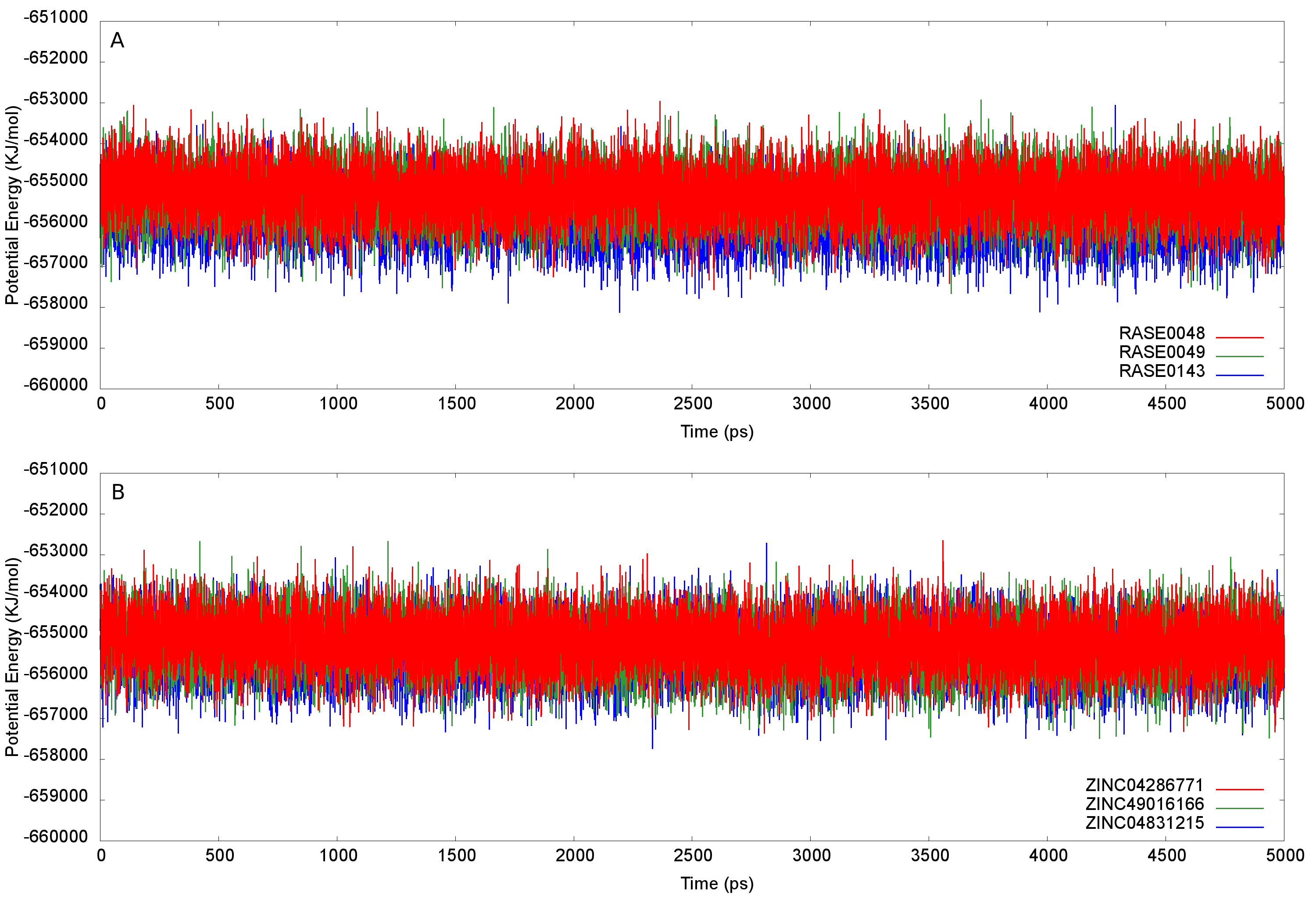

Supplement: Figure S1 — Potential energy profiles of best PDMs and representative ZINC leads. Potential energies of complexes, as a function of time: (A) Best PDMs and (B) Representative molecules from analogs of PDM leads. (TIFF) [file pone.0061327.s001.tiff]

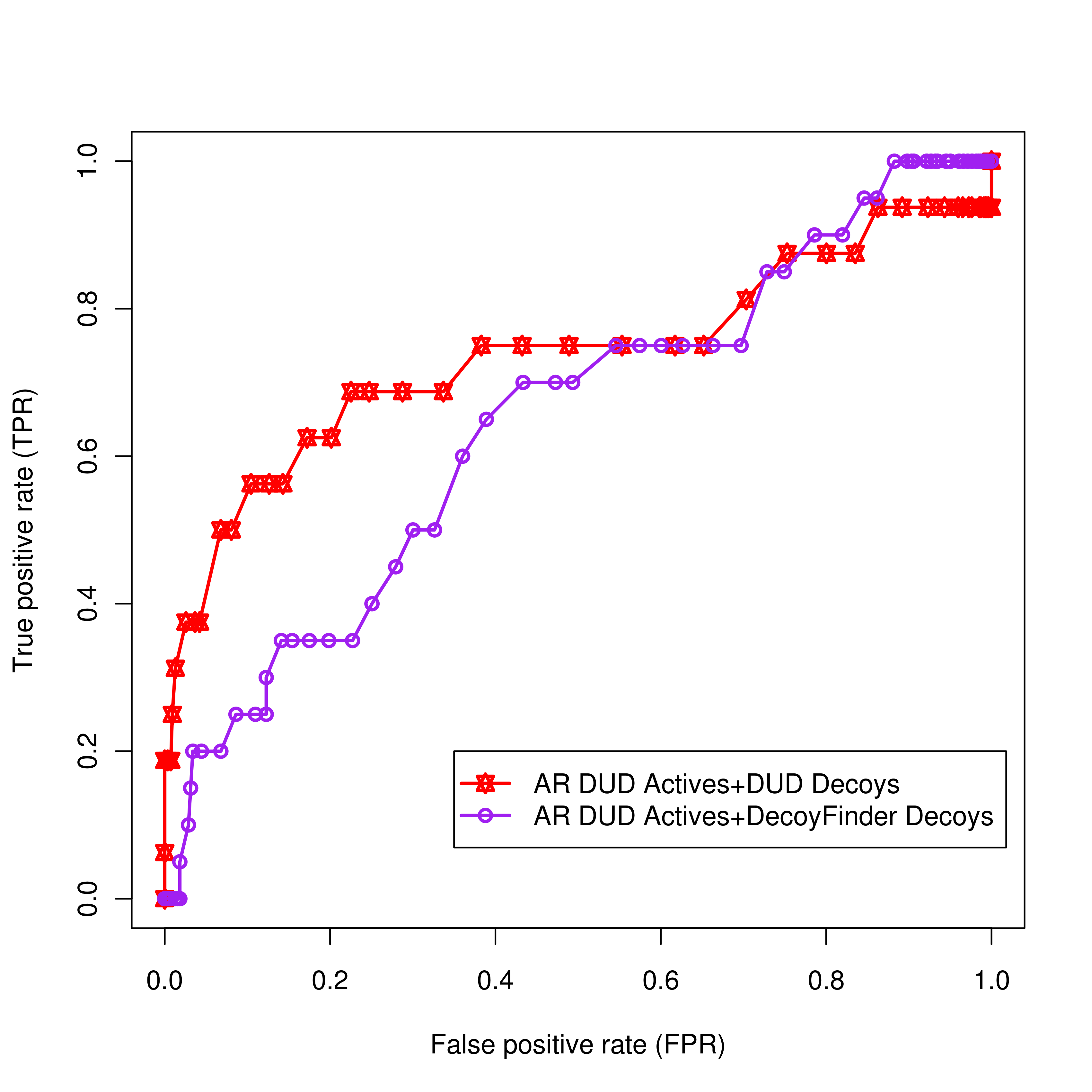

Supplement: Figure S2 — ROC analysis to evaluate reliability of DecoyFinder decoys. Comparison of results from ROC curve analysis for the AR DUD actives against (A) AR DUD decoys and (B) AR DecoyFinder decoys. The AUC for the former was 0.74, whereas that for the latter was 0.64, asserting the reliability of DecoyFinder. (TIFF) [file pone.0061327.s002.tiff]
