# Supplementary material for: Prospecting for Novel Plant-Derived Molecules of Rauvolfia serpentina as Inhibitors of Aldose Reductase, a Potent Drug Target for Diabetes and Its Complications
Source: PLoS One. 2013 Apr 17;8(4):e61327. doi: 10.1371/journal.pone.0061327 (PMC3629236; doi:10.1371/journal.pone.0061327)
Supplement: Table S1 — A structured dataset of R. serpentina PDMs. (PDF) [file pone.0061327.s003.pdf]

Table S1. A structured dataset of *R. serpentina* PDMs. This PDM dataset have 147 compounds and additional details including chemical name, plant part, IUPAC name and 2D structure.

PDM_ID	PDM_NAME	PLANT PART	IUPAC	STRUCTURE
RASE0001 [1,3]	Reserpine	Stem, Leaves, Roots, Root bark	(1R,15S,17R,18R,19S,20S)-6,18-dimethoxy-17-(3,4,5-trimethoxybenzoyl)oxy-1,3,11,12,14,15,16,17,18,19,20,21-dodecahydroyohimban-19-carboxylate	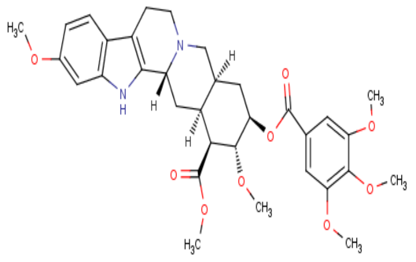
RASE0002 [2,4,5]	Ajmaline	Stem, Leaves, Roots, Root bark	(1R,9R,10S,13R,14R,16S,18S)-13-ethyl-8-methyl-8,15-diazahexacyclo [14.2.1.0^1,9.0^2,7.0^10,15.0^12,17] nonadeca-2(7),3,5-triene-14,18-diol	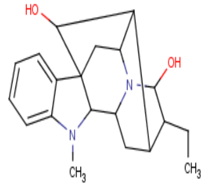

RASE0003<br><br>[6]	Isoajmaline	Stem, Leaves, Roots, Root bark	13-ethyl-8-methyl-8,15-diazahexacyclo[14.2.1.0^{1,9}.0^{2,7}.0^{10,15}.0^{12,17}]nonadeca-2(7),3,5-triene-14,18-diol	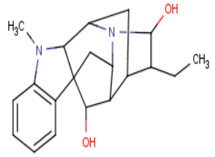
RASE0004<br><br>[6]	Neoajmaline	Stem, Leaves, Roots, Root bark	3-Ethyl-4,8-dihydroxy-13-methyl-5-propyl-1,3,4,7,8,13,13a,13b-octahydro-2H,6H-2,7-cyclo-6,8a-methano-pyrido[1',2':1,2]azepino[3,4-b]indol-5-ium	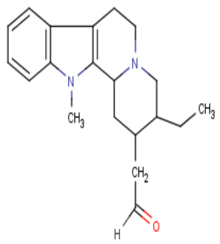
RASE0005<br><br>[2,7]	Ajmalicine	Stem, Leaves, Roots, Root bark	(19α)-16,17-didehydro- 19-methyloxayohimban- 16-carboxylic acid methyl ester	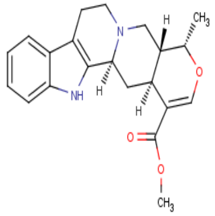

RASE0006<br><br>[6]	Raubasine	Stem, Leaves, Roots, Root bark	Methyl (1S,15R,16S,20S)-16-methyl-17-oxa-3,13-diazapentacyclo[11.8.0.0^{2,10}.0^{4,9}.0^{15,20}]henicosa-2(10),4,6,8,18-pentaene-19-carboxylate	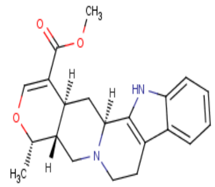
RASE0007<br><br>[7]	Yohimbine	Stem, Leaves, Roots, Root bark	(1S,15R,18S,19R,20S)-18-hydroxy-1,3,11,12,14,15,16,17,18,19,20,21-dodecahydroyohimban-19-carboxylate	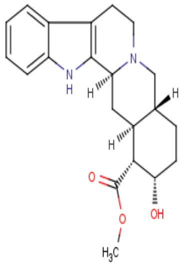
RASE0008<br><br>[3]	Deserpidine	Stem, Leaves, Roots, Root bark	Methyl (1R,15S,17R,18R,19S,20S)-18-methoxy-17-(3,4,5-trimethoxybenzoyloxy)-3,13-diazapentacyclo[11.8.0.0^{2,10}.0^{4,9}.0^{15,20}]henicosa-2(10),4,6,8-tetraene-19-carboxylate	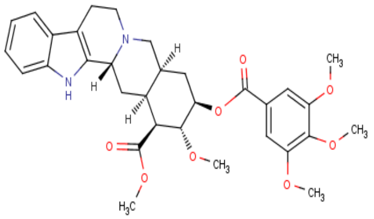

RASE0009<br><br>[6,7]	Rescinnamine	Stem, Leaves, Roots, Root bark	Methyl (1R,15S,17R,18R,19S,20S)-6,18-dimethoxy-17-{[(2E)-3-(3,4,5-trimethoxyphenyl)prop-2-enoyl]oxy}-3,13-diazapentacyclo[11.8.0.0^{2,10}.0^{4,9}.0^{15,20}]henicosa-2(10),4(9),5,7-tetraene-19-carboxylate	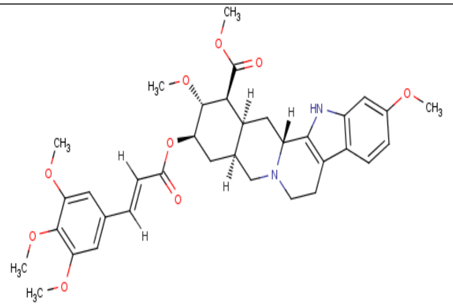
RASE0010<br><br>[6]	Serpentinine	Stem, Leaves, Roots, Root bark	11-{2-[(3Z)-3-ethylidene-1H,2H,3H,4H,6H,7H,12H,12bH-indolo[2,3-a]quinolizin-2-yl]-3-methoxy-3-oxopropyl}-19-(methoxycarbonyl)-16-methyl-17-oxa-3,13$l^{5}-diazapentacyclo[11.8.0.0^{2,10}.0^{4,9}.0^{15,20}]henicosa-1(13),2(10),4,6,8,11,18-heptaen-13-ylium	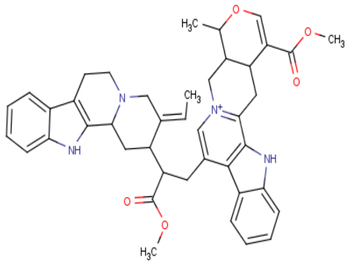
RASE0011<br><br>[8]	Corynanthine	Stem, Leaves, Roots, Root bark	Methyl (1S,15R,18S,19S,20S)-18-hydroxy-3,13-diazapentacyclo[11.8.0.0^{2,10}.0^{4,9}.0^{15,20}]henicosa-2(10),4,6,8-tetraene-19-carboxylate	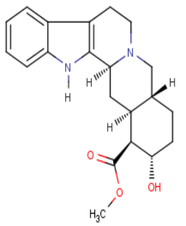

RASE0012<br><br>[8]	Papaverine	Stem, Leaves, Roots, Root bark	1-[(3,4-dimethoxyphenyl)methyl]-6,7-dimethoxyisoquinoline	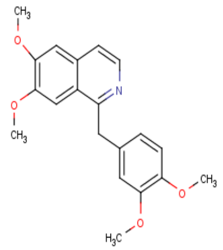
RASE0013<br><br>[4]	Sarpagine	Stem, Leaves, Roots, Root bark	(1S,12S,13R,14S,15E)-15-ethylidene-13-(hydroxymethyl)-3,17-diazapentacyclo[12.3.1.0^{2,10}.0^{4,9}.0^{12,17}]octadeca-2(10),4(9),5,7-tetraen-7-ol	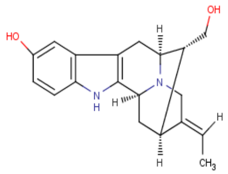
RASE0014<br><br>[9,10]	Serpentine	Stem, Leaves, Roots, Root bark	(15R,16S,20S)-19-(methoxycarbonyl)-16-methyl-17-oxa-3,13$l^{5}-diazapentacyclo[11.8.0.0^{2,10}.0^{4,9}.0^{15,20}]henicosa-1(13),2(10),4,6,8,11,18-heptaen-13-ylium	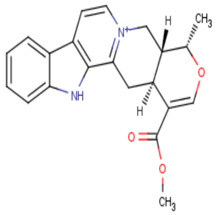
RASE0015<br><br>[8]	Serpinine	Stem, Leaves, Roots, Root bark	(1R,9R,10S,12R,13E,16S,17R,18R)-13-ethylidene-8-methyl-8,15-diazahexacyclo[14.2.1.0^{1,9}.0^{2,7}.0^{10,15}.0^{12,17}]nonadeca-2,4,6-trien-18-ol	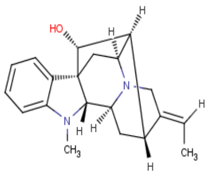

RASE0016<br><br>[6]	Alstonine	Roots	(15S,16S,20S)-19-(methoxycarbonyl)-16-methyl-17-oxa-3,13$l^{5}-diazapentacyclo[11.8.0.0^{2,10}.0^{4,9}.0^{15,20}]henicosa-1(13),2(10),4(9),5,7,11,18-heptaen-13-ylium-3-ide	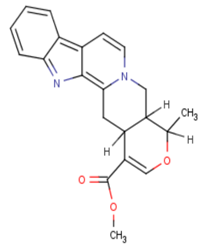
RASE0017<br><br>[8]	Ajmalinine	Stem, Leaves, Roots, Root bark	US	
RASE0018<br><br>[8,11]	Chandrine	Stem, Leaves, Roots, Root bark	US	
RASE0019<br><br>[11]	Renoxidine	Roots	Methyl 6,18-dimethoxy-13-oxo-17-(3,4,5-trimethoxybenzoyloxy)-3,13$l^{5}-diazapentacyclo[11.8.0.0^{2,10}.0^{4,9}.0^{15,20}]henicosa-2(10),4,6,8-tetraene-19-carboxylate	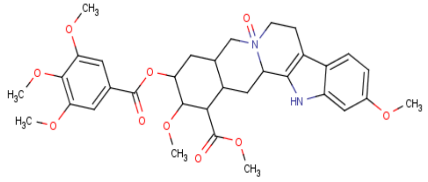

RASE0020<br><br>[11]	Reserpiline	Stem, Leaves, Roots, Root bark	Methyl (1R,15S,16S,20S)-6,7-dimethoxy-16-methyl-17-oxa-3,13-diazapentacyclo[11.8.0.0^{2,10}.0^{4,9}.0^{15,20}]henicosa-2(10),4,6,8,18-pentaene-19-carboxylate	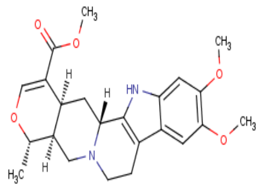
RASE0021<br><br>[11,12]	Reserpinine	Stem, Leaves, Roots, Root bark	Methyl(1R,15S,17R,18R,19S,20S)-6,18-dimethoxy-17-[(E)-3-(3,4,5-trimethoxyphenyl)prop-2-enoyl]oxy-1,3,11,12,14,15,16,17,18,19,20,21-dodecahydroyohimban-19-carboxylate	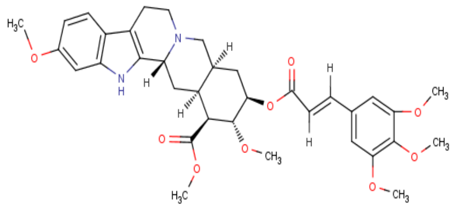
RASE0022<br><br>[11]	Ophioxylin	Roots	5-hydroxy-2-methyl-1,4-dihydronaphthalene-1,4-dione	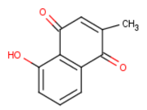
RASE0023<br><br>[13]	Rauwolscine	Roots	Methyl (1S,15S,18S,19S,20S)-18-hydroxy-3,13-diazapentacyclo[11.8.0.0^{2,10}.0^{4,9}.0^{15,20}]henicosa-2(10),4,6,8-tetraene-19-carboxylate	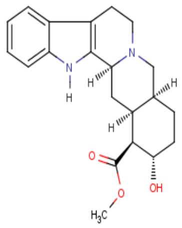

RASE0024<br><br>[8]	Thebaine	Roots	(1S,5R,13R)-10,14-dimethoxy-4,5-dimethyl-12-oxa-4-azapentacyclo[9.6.1.0^{1,13}.0^{5,17}.0^{7,18}]octadeca-7(18),8,10,14,16-pentaene	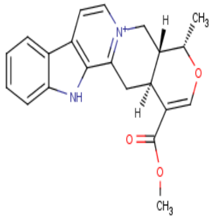
RASE0025<br><br>[14]	7-Dehydrositosterol	Roots	(1S,2R,5S,11R,15R)-14-[(2R,5S)-5-ethyl-6-methylheptan-2-yl]-2,15-dimethyltetracyclo[8.7.0.0^{2,7}.0^{11,15}]heptadeca-7,9-dien-5-ol	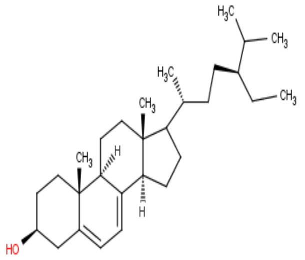
RASE0026<br><br>[15,16]	Stigmasterol	Leaves	(3S,8S,9S,10R,13R,14S,17R)-17-[(E,2R,5S)-5-ethyl-6-methylhept-3-en-2-yl]-10,13-dimethyl-2,3,4,7,8,9,11,12,14,15,16,17-dodecahydro-1H-cyclopenta[a]phenanthren-3-ol	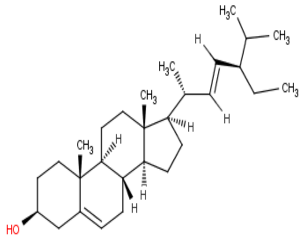
RASE0027<br><br>[11]	Starch	Roots	US	

RASE0028<br><br>[15,17]	2,6-Dimethoxybenzoquinone	Roots	2,6-dimethoxycyclohexa-2,5-diene-1,4-dione	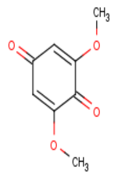
RASE0029<br><br>[8,12]	Tetraphyllicine	Leaves, Root Bark	(1R,9R,10S,12R,13E,16S,17R,18R)-13-ethylidene-8-methyl-8,15-diazahexacyclo[14.2.1.0^{1,9}.0^{2,7}.0^{10,15}.0^{12,17}]nonadeca-2,4,6-trien-18-ol	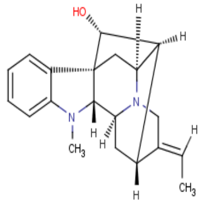
RASE0030<br><br>[7,12,18,19]	Raucaffricine	Roots, Hybrid Cell Culture, Cell Suspension Culture	13-ethylidene-14-{[3,4,5-trihydroxy-6-(hydroxymethyl)oxan-2-yl]oxy}-8,15-diazahexacyclo[14.2.1.0^{1,9}.0^{2,7}.0^{10,15}.0^{12,17}]nonadeca-2,4,6,8-tetraen-18-yl acetate	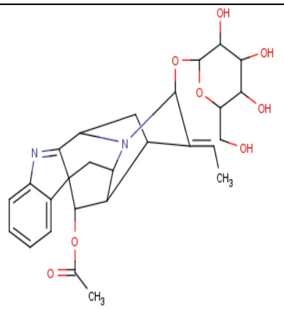

RASE0031<br><br>[1,4,5,7,18]	Vomilenine	Root culture	(1R,10S,12R,13E,14R,16S)-13-ethylidene-14-hydroxy-8,15-diazahexacyclo[14.2.1.0^{1,9}.0^{2,7}.0^{10,15}.0^{12,17}]nonadeca-2,4,6,8-tetraen-18-yl acetate	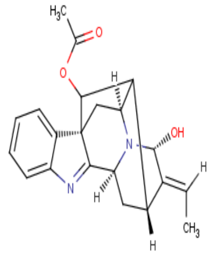
RASE0032<br><br>[20]	10-Hydroxy-N(α)-demethyl-19,20-dehydroraumacline	Root culture	US	
RASE0033<br><br>[21,22]	Raumacline	Root culture	(1S,12S,13S,18S)-1,11,12,13,18-pentahydrogenio-17-(1-hydrogenioethyl)-3-methyl-15-oxa-3,20-diazapentacyclo[10.7.1.0^{2,10}.0^{4,9}.0^{13,18}]icosa-2(10),4(9),5,7-tetraen-14-ol	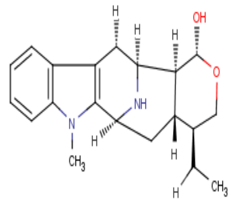

RASE0034<br><br>[4,5,23]	Raucaffrinoline	Cell culture	(1R,10S,12S,13R,14S,16S)-13-(hydroxymethyl)-14-methyl-8,15-diazahexacyclo[14.2.1.0^{1,9}.0^{2,7}.0^{10,15}.0^{12,17}]nonadeca-2,4,6,8-tetraen-18-yl acetate	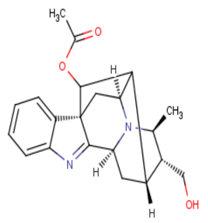
RASE0035<br><br>[4,5,7,12,23]	Perakine	Hybrid Cell Culture	(1R,10S,13R,14S,16S,18R)-13-formyl-14-methyl-8,15-diazahexacyclo[14.2.1.0^{1,9}.0^{2,7}.0^{10,15}.0^{12,17}]nonadeca-2,4,6,8-tetraen-18-yl acetate	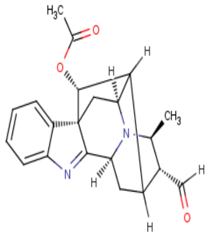
RASE0036<br><br>[4,5,18]	Vinorine	Cell culture	(1R,10S,16S)-13-ethylidene-8,15-diazahexacyclo[14.2.1.0^{1,9}.0^{2,7}.0^{10,15}.0^{12,17}]nonadeca-2,4,6,8-tetraen-18-yl acetate	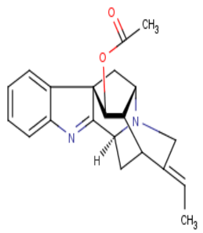

RASE0037<br><br>[5,24]	16-epi-vellosimine	Cell culture	(1S,12S,14S)-15-ethylidene-3,17-diazapentacyclo[12.3.1.0^{2,10}.0^{4,9}.0^{12,17}]octadeca-2(10),4(9),5,7-tetraene-13-carbaldehyde	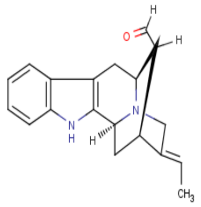
RASE0038<br><br>[25]	11-Methoxyvinorine	Cell culture	(1R,10S,12R,13E,16S,18R)-13-ethylidene-5-methoxy-8,15-diazahexacyclo[14.2.1.0^{1,9}.0^{2,7}.0^{10,15}.0^{12,17}]nonadeca-2(7),3,5,8-tetraen-18-yl acetate	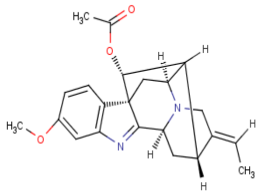
RASE0039<br><br>[4]	Vellosimine	Cell culture	(12S,14S,15E)-15-ethylidene-3,17-diazapentacyclo[12.3.1.0^{2,10}.0^{4,9}.0^{12,17}]octadeca-2(10),4,6,8-tetraene-13-carbaldehyde	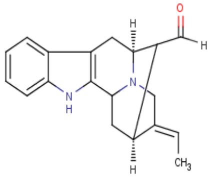
RASE0040<br><br>[5]	1,2-dihydrovomilenine	Cell culture	(1R,9R,10S,12R,13E,14R,16S,18R)-13-ethylidene-14-hydroxy-8,15-diazahexacyclo[14.2.1.0^{1,9}.0^{2,7}.0^{10,15}.0^{12,17}]nonadeca-2,4,6-trien-18-yl acetate	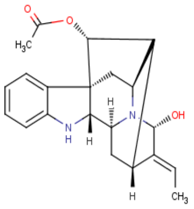

RASE0041<br><br>[5,7]	17-O-Acetyl-norajmaline	Cell culture	(1R,9R,10S,12R,13S,14R,16S)-13-ethyl-14-hydroxy-8-methyl-8,15-diazahexacyclo[14.2.1.0^{1,9}.0^{2,7}.0^{10,15}.0^{12,17}]nonadeca-2,4,6-trien-18-yl acetate	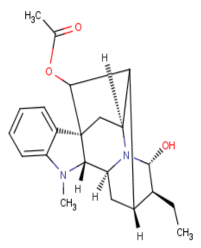
RASE0042<br><br>[4,5,12]	Norajmaline	Hybrid Cell Culture	(1R,9R,10S,12R,13S,14R,16S)-13-ethyl-8-methyl-8,15-diazahexacyclo[14.2.1.0^{1,9}.0^{2,7}.0^{10,15}.0^{12,17}]nonadeca-2,4,6-triene-14,18-diol	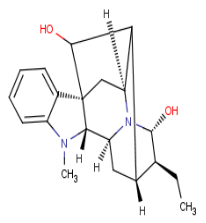
RASE0043<br><br>[26]	Rauwolfine	Stem, Leaves, Roots, Root bark	(9R,14R)-13-ethyl-8-methyl-8,15-diazahexacyclo[14.2.1.0^{1,9}.0^{2,7}.0^{10,15}.0^{12,17}]nonadeca-2,4,6-triene-14,18-diol	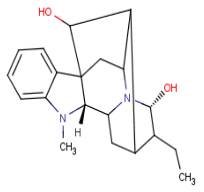
RASE0044<br><br>[6,8]	Rauwolfinine	Root Bark	13-ethyl-8-methyl-19-oxa-8,15-diazahexacyclo[14.3.1.0^{1,9}.0^{2,7}.0^{10,15}.0^{12,17}]icosa-2(7),3,5-trien-18-ol	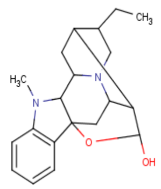

RASE0045<br><br>[27]	Rescinnamidine	Roots	Methyl (1R,15S,17R,18R,19S,20S)-6,18-dimethoxy-17-{[3-(3,4,5-trimethoxyphenyl)propanoyl]oxy}-3,13-diazapentacyclo[11.8.0.0^{2,10}.0^{4,9}.0^{15,20}]henicosa-2(10),4(9),5,7-tetraene-19-carboxylate	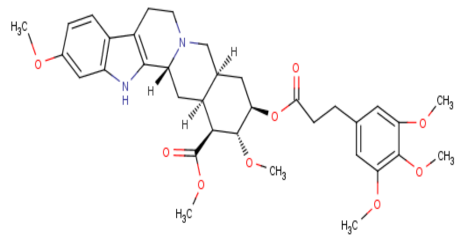
RASE0046<br><br>[27]	Rescinnaminol	Roots	1-({7-methoxy-1,10-diazapentacyclo[11.8.0.0^{3,11}.0^{4,9}.0^{15,20}]henicosa-3(11),4(9),5,7-tetraen-18-yl}oxy)-3-(3,4,5-trimethoxyphenyl)propan-1-ol	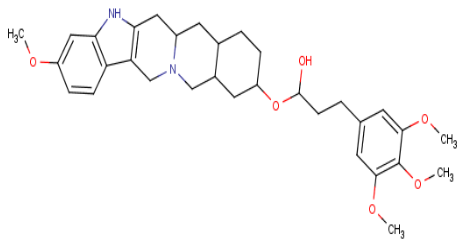
RASE0047<br><br>[6]	Tetraphylline	Stem, Leaves, Roots, Root bark	Methyl (1S,15R,16S,20S)-6-methoxy-16-methyl-17-oxa-3,13-diazapentacyclo[11.8.0.0^{2,10}.0^{4,9}.0^{15,20}]henicosa-2(10),4(9),5,7,18-pentaene-19-carboxylate	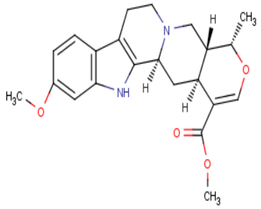

RASE0048<br><br>[27]	Indobine	Roots	Benzyl 3-(1H-indol-3-yl)propanoate	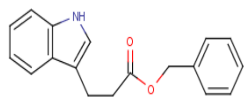
RASE0049<br><br>[8]	Indobinine	Roots	Cyclohexyl 3-(1H-indol-3-yl)propanoate	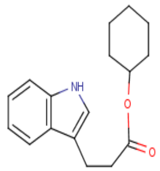
RASE0050<br><br>[8,12]	Isorauhimbine	Stem, Leaves, Roots, Root bark	Methyl (1R,15S,18S,19S,20S)-18-hydroxy-3,13-diazapentacyclo[11.8.0.0^{2,10}.0^{4,9}.0^{15,20}]henicosa-2(10),4,6,8-tetraene-19-carboxylate	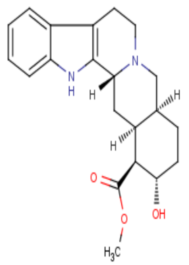

RASE0051<br><br>[16,17]	Rauhimbine	Stem, Leaves, Roots, Root bark	Methyl (1S,15R,18S,19S,20S)-18-hydroxy-3,13-diazapentacyclo[11.8.0.0^{2,10}.0^{4,9}.0^{15,20}]henicosa-2(10),4,6,8-tetraene-19-carboxylate	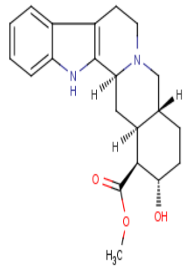
RASE0052<br><br>[8]	Sandwicolidine	Undried roots	14-ethyl-13-methoxy-8-methyl-8,15-diazahexacyclo[14.2.1.0^{1,9}.0^{2,7}.0^{10,15}.0^{12,17}]nonadeca-2(7),3,5-trien-18-ol	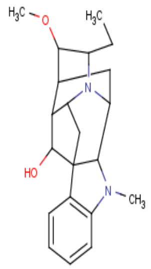
RASE0053<br><br>[8]	Sandwicoline	Undried roots	14-(1-hydroxybutan-2-yl)-3,16-dimethyl-3,16-diazapentacyclo[10.3.1.1^{10,13}.0^{2,10}.0^{4,9}]heptadeca-4(9),5,7-trien-17-ol	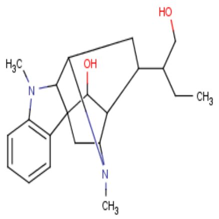

RASE0054<br><br>[8]	Yohambinine	Roots	12-methyl-3,13-diazapentacyclo[11.8.0.0^{2,10}.0^{4,9}.0^{15,20}]henicosa-2(10),4(9),5,7-tetraene	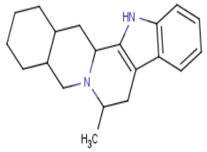
RASE0055<br><br>[28]	Sucrose	US	(2R,3R,4S,5S,6R)-2-[(2S,3S,4S,5R)-3,4-dihydroxy-2,5-bis(hydroxymethyl)oxolan-2-yl]oxy-6-(hydroxymethyl)oxane-3,4,5-triol	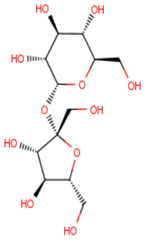
RASE0056<br><br>[29]	Oleic acid	US	(9Z)-octadec-9-enoic acid	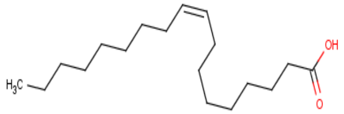

RASE0057<br><br>[29]	Fumaric acid	US	(2E)-but-2-enedioic acid	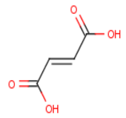
RASE0058<br><br>[29]	γ-Sitosterol	US	(3S)-17-[(5S)-5-ethyl-6-methylheptan-2-yl]-10,13-dimethyl-2,3,4,7,8,9,11,12,14,15,16,17-dodecahydro-1H-cyclopenta[a]phenanthren-3-ol (3S)-17-[(5S)-5-ethyl-6-methylheptan-2-yl]-10,13-dimethyl-2,3,4,7,8,9, 11,12,14,15,16,17-dodecahydro-1H-cyclopenta[a]phenanthren-3-o	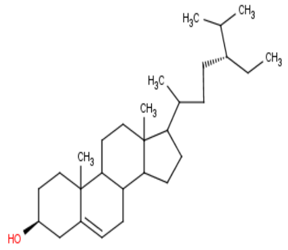
RASE0059<br><br>[29]	β-Sitosterol	US	(3S,8S,9S,10R,13R,14S,17R)-17-[(2S,5S)-5-ethyl-6-methylheptan-2-yl]-10,13-dimethyl-2,3,4,7,8,9,11,12,14,15,16,17-dodecahydro-1H-cyclopenta[a]phenanthren-3-ol	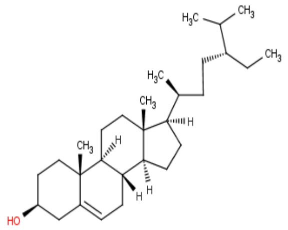

RASE0060<br><br>[30]	Serposterol	US	14-[(3E)-5-ethyl-6-methylhept-3-en-2-yl]-2,15-dimethyltetracyclo[8.7.0.0^{2,7}.0^{11,15}]heptadec-7-en-5-ol	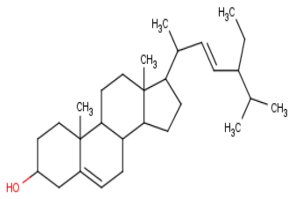
RASE0061<br><br>[28]	Diisobutyl phthalate	Roots	1,2-bis(2-methylpropyl) benzene-1,2-dicarboxylate	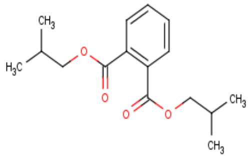
RASE0062<br><br>[3]	Deserpidic acid lactone	Dried roots	(1S,2S,4R,18S,20R,23R)-23-methoxy-21-oxa-6,16-diazahexacyclo[18.2.1.0^{2,18}.0^{4,16}.0^{5,13}.0^{7,12}]tricosa-5(13),7(12),8,10-tetraen-22-one	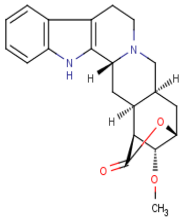
RASE0063<br><br>[4]	Vallesiachotamine	Hairy Root Culture	Methyl(2S,12bS)-2-[(E)-1-oxobut-2-en-2-yl]-1,2,6,7,12,12b-hexahydroindolo[2,3-a]quinolizine-3-carboxylate	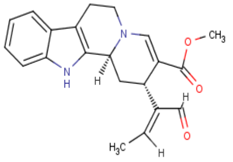

RASE0064<br><br>[22]	19-Hydroxy-Nβ-methylraumacline	Cell Suspension Culture	(1S,12S,13S,14R,17S,18S)-17-(1-hydroxyethyl)-3,20-dimethyl-15-oxa-3,20-diazapentacyclo[10.7.1.0^{2,10}.0^{4,9}.0^{13,18}]icosa-2(10),4(9),5,7-tetraen-14-ol	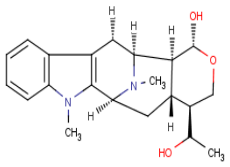
RASE0065<br><br>[22]	6α-Hydroxyraumacline	Cell Suspension Culture	(1S,11S,12R,13S,14R,17S,18S)-17-ethyl-3-methyl-15-oxa-3,20-diazapentacyclo[10.7.1.0^{2,10}.0^{4,9}.0^{13,18}]icosa-2(10),4(9),5,7-tetraene-11,14-diol	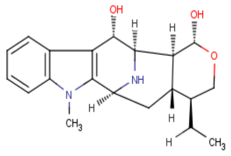
RASE0066<br><br>[22]	6α-Methoxyraumacline	Cell Suspension Culture	(1S,11S,12R,13S,14R,17S,18S)-17-ethyl-11-methoxy-3-methyl-15-oxa-3,20-diazapentacyclo[10.7.1.0^{2,10}.0^{4,9}.0^{13,18}]icosa-2(10),4(9),5,7-tetraen-14-ol	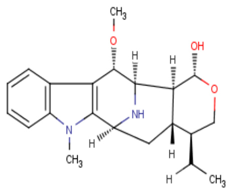
RASE0067<br><br>[12]	N(b)-Methylajmaline	Dried roots	(1R,9R,12S,16S)-13-ethyl-14,18-dihydroxy-8,15-dimethyl-8,15-diazahexacyclo[14.2.1.0^{1,9}.0^{2,7}.0^{10,15}.0^{12,17}]nonadeca-2(7),3,5-trien-15-ium	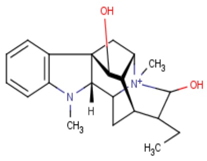

RASE0068<br><br>[12]	N(b)-Methylisoajmaline	Dried roots	(1R,9R,12S,13R,14S,16S)-13-ethyl-14,18-dihydroxy-8,15-dimethyl-8,15-diazahexacyclo[14.2.1.0^{1,9}.0^{2,7}.0^{10,15}.0^{12,17}]nonadeca-2(7),3,5-trien-15-ium	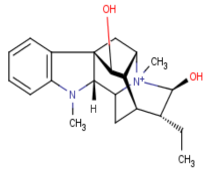
RASE0069<br><br>[12]	3-Hydroxysarpagine	Dried roots	(1R,12S,14S)-15-ethylidene-13-(hydroxymethyl)-3,17-diazapentacyclo[12.3.1.0^{2,10}.0^{4,9}.0^{12,17}]octadeca-2(10),4(9),5,7-tetraene-1,7-diol	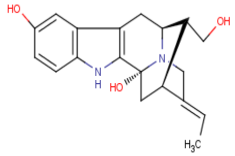
RASE0070<br><br>[12]	Yohimbinic acid	Dried roots	(1S,15R,18S,19R,20S)-18-hydroxy-3,13-diazapentacyclo[11.8.0.0^{2,10}.0^{4,9}.0^{15,20}]henicosa-2(10),4,6,8-tetraene-19-carboperoxoic acid	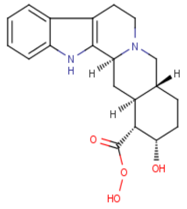
RASE0071<br><br>[12]	Isorauhimbinic acid	Dried roots	(1S,15R,18S,19R,20S)-18-hydroxy-3,13-diazapentacyclo[11.8.0.0^{2,10}.0^{4,9}.0^{15,20}]henicosa-2(10),4,6,8-tetraene-19-carboperoxoic acid	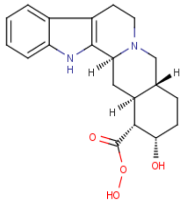

RASE0072<br><br>[12]	7-Epiloganin	Dried roots	Methyl (1R,2R,3aS,7aS)-2-hydroxy-1-methyl-7-{[3,4,5-trihydroxy-6-(hydroxymethyl)oxan-2-yl]oxy}-2,3,3a,6,7,7a-hexahydro-1H-indene-4-carboxylate	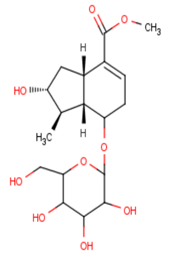
RASE0073<br><br>[12]	6′-O-(3,4,5-trimethoxybenzoyl)glomeratose A	Dried roots	(3,4,5-trihydroxy-6-{[4-hydroxy-2,5-bis(hydroxymethyl)-3-{[(2E)-3-(3,4,5-trimethoxyphenyl)prop-2-enoyl]oxy}oxolan-2-yl]oxy}oxan-2-yl)methyl 3,4,5-trimethoxybenzoate	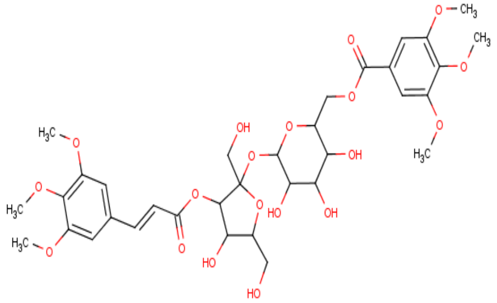
RASE00074<br><br>[12]	Normacusine B	Dried roots	[(15Z)-15-ethylidene-3,17-diazapentacyclo[12.3.1.0^{2,10}.0^{4,9}.0^{12,17}]octadeca-2(10),4,6,8-tetraen-13-yl]methanol	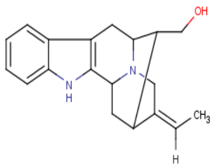

RASE0075<br><br>[12]	Geissoschizol	Dried roots	2-[(2S,4R,5E)-5-ethylidene-7,17-diazatetracyclo[8.7.0.0^{2,7}.0^{11,16}]heptadeca-1(10),11(16),12,14-tetraen-4-yl]ethan-1-ol	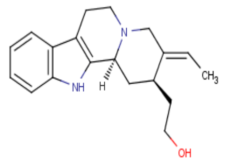
RASE0076<br><br>[12]	Rhazimanine	Dried roots	Methyl (11S,12E,18S)-12-ethylidene-17-oxo-14-azapentacyclo[9.5.3.0^{1,10}.0^{2,7}.0^{14,18}]nonadeca-2,4,6,8-tetraene-10-carboxylate	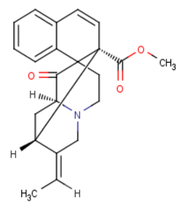
RASE0077<br><br>[12]	18-Hydroxyepiallo yohimbine	Dried roots	Methyl 17,18-dihydroxy-3,13-diazapentacyclo[11.8.0.0^{2,10}.0^{4,9}.0^{15,20}]henicosa-2(10),4,6,8-tetraene-19-carboxylate	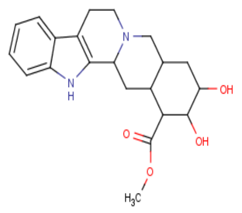
RASE0078<br><br>[3,8,12]	Methyl reserpate	Dried roots	Methyl 17-hydroxy-6,18-dimethoxy-3,13-diazapentacyclo[11.8.0.0^{2,10}.0^{4,9}.0^{15,20}]henicosa-2(10),4(9),5,7-tetraene-19-carboxylate	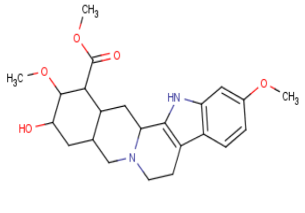

RASE0079<br><br>[12]	Loganic acid	Dried roots	(1S,4aS,6S,7R,7aS)-6-hydroxy-7-methyl-1-{[(2S,3R,4S,5S,6R)-3,4,5-trihydroxy-6-(hydroxymethyl)oxan-2-yl]oxy}-1H,4aH,5H,6H,7H,7aH-cyclopenta[c]pyran-4-carboxylic acid	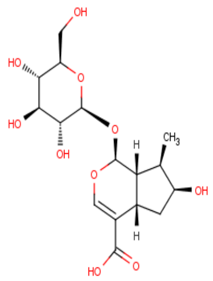
RASE0080<br><br>[12]	7-Deoxyloganic acid	Dried roots	7-methyl-1-{[3,4,5-trihydroxy-6-(hydroxymethyl)oxan-2-yl]oxy}-1H,4aH,5H,6H,7H,7aH-cyclopenta[c]pyran-4-carboxylic acid	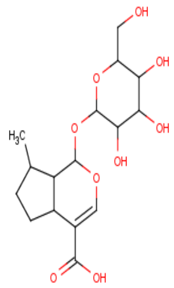
RASE0081<br><br>[12]	Secoxyloganin	Dried roots	2-[(2S,3R,4S)-3-ethenyl-5-(methoxycarbonyl)-2-{[(2S,3R,4S,5S,6R)-3,4,5-trihydroxy-6-(hydroxymethyl)oxan-2-yl]oxy}-3,4-dihydro-2H-pyran-4-yl]acetic acid	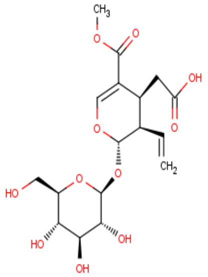
RASE0082<br><br>[12]	(+)-Isolariciresinol 3a-O-beta-D-glucopyranoside	Dried roots	US	

RASE0083<br><br>[12]	Glomeratose A	Dried roots	4-hydroxy-2,5-bis(hydroxymethyl)-2-{[3,4,5-trihydroxy-6-(hydroxymethyl)oxan-2-yl]oxy}oxolan-3-yl (2E)-3-(3,4,5-trimethoxyphenyl)prop-2-enoate	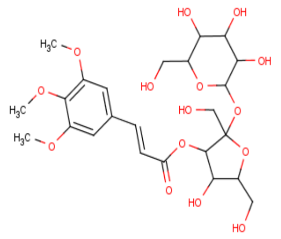
RASE0084<br><br>[12]	16-Epinormacusine B	Dried roots	[(15E)-15-ethylidene-3,17-diazapentacyclo[12.3.1.0^{2,10}.0^{4,9}.0^{12,17}]octadeca-2(10),4(9),5,7-tetraen-13-yl]methanol	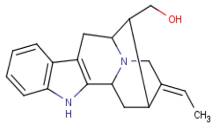
RASE0085<br><br>[12]	Swertiaside	Dried roots	6-(3-hydroxybenzoyloxy)-7-methyl-1-{[3,4,5-trihydroxy-6-(hydroxymethyl)oxan-2-yl]oxy}-1H,4aH,5H,6H,7H,7aH-cyclopenta[c]pyran-4-carboxylic acid	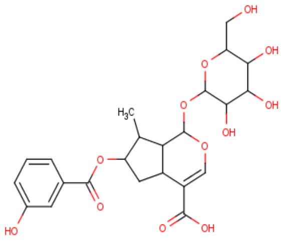
RASE0086<br><br>[9]	3,4,5,6-Tetradehydroyohimbine	Roots	(15R,18S,19R,20S)-18-hydroxy-19-(methoxycarbonyl)-3,13$l^{5}-diazapentacyclo[11.8.0.0^{2,10}.0^{4,9}.0^{15,20}]henicosa-1(13),2(10),4,6,8,11-hexaen-13-ylium-3-ide	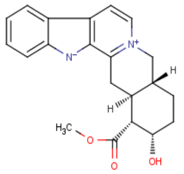

RASE0087<br><br>[9]	3,4,5,6-Tetradehydro-(Z)-geissoschizol	Roots	(2R,3Z)-3-ethylidene-2-(2-hydroxyethyl)-1H,2H,3H,4H,12H-5$l^{5},12-indolo[2,3-a]quinolizin-5-ylium-12-ide	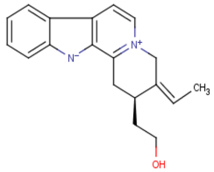
RASE0088<br><br>[9]	3,4,5,6-Tetradehydrogeissoschizol	Roots	(2R,3E)-3-ethylidene-2-(2-hydroxyethyl)-1H,2H,3H,4H,12H-5$l^{5},12-indolo[2,3-a]quinolizin-5-ylium-12-ide	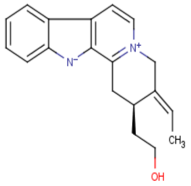
RASE0089<br><br>[9]	3,4,5,6-Tetradehydrogeissoschizine-17-O-b-d-glucopyranoside	Roots	(2S,3E)-3-ethylidene-2-[(1Z)-3-methoxy-3-oxo-1-{[3,4,5-trihydroxy-6-(hydroxymethyl)oxan-2-yl]oxy}prop-1-en-2-yl]-1H,2H,3H,4H,12H-5$l^{5},12-indolo[2,3-a]quinolizin-5-ylium-12-ide	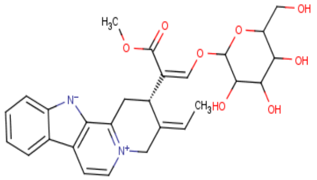
RASE0090<br><br>[7]	3-Oxo-rhazinilam	Hybrid Cell Culture	12-ethyl-8,16-diazatetracyclo[10.6.1.0^{2,7}.0^{16,19}]nonadeca-1(19),2(7),3,5,17-pentaene-9,15-dione	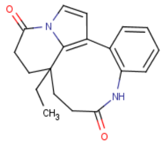

RASE0091<br><br>[31,32]	Arbutin	Cell Suspension Culture	(2R,3S,4S,5R,6S)-2-(hydroxymethyl)-6-(4-hydroxyphenoxy)oxane-3,4,5-triol	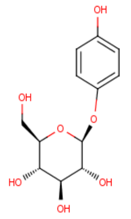
RASE0092<br><br>[33]	Ajmalimine	Roots	13-ethyl-14-hydroxy-8-methyl-8,15-diazahexacyclo[14.2.1.0^{1,9}.0^{2,7}.0^{10,15}.0^{12,17}]nonadeca-2,4,6-trien-18-yl 3,4,5-trimethoxybenzoate	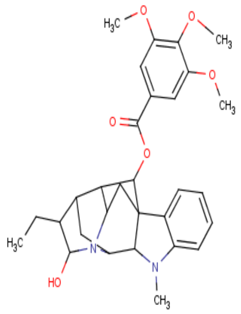
RASE0093<br><br>[5,24]	Tryptamine	Cell culture	2-(1H-indol-3-yl)ethanamine	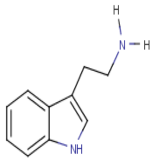

RASE0094<br><br>[5,24]	Secologanin	Cell culture	Methyl (2S,3R,4S)-3-ethenyl-4-(2-oxoethyl)-2-{[(2S,3R,4S,5S,6R)-3,4,5-trihydroxy-6-(hydroxymethyl)oxan-2-yl]oxy}-3,4-dihydro-2H-pyran-5-carboxylate	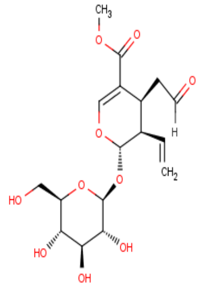
RASE0095<br><br>[22]	Nβ-Methylraumacline	Cell Suspension Culture	(1S,12S,13S,14R,17S,18S)-17-ethyl-3,20-dimethyl-15-oxa-3,20-diazapentacyclo[10.7.1.0^{2,10}.0^{4,9}.0^{13,18}]icosa-2(10),4(9),5,7-tetraen-14-ol	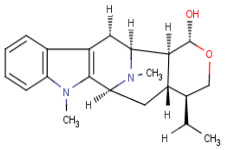
RASE0096<br><br>[4,7]	Tetrahydroalstonine	Hairy Root culture	(19α)-16,17-didehydro- 19-methyloxayohimban- 16-carboxylic acid methyl ester	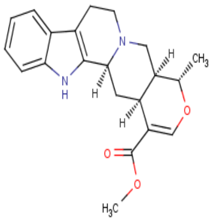

RASE0097<br><br>[4,5,23]	19(S),20(R)-dihydroperaksine	Hairy Root culture	[(1S,12S,13R,15R,16S)-15-(hydroxymethyl)-16-methyl-3,17-diazapentacyclo[12.3.1.0^{2,10}.0^{4,9}.0^{12,17}]octadeca-2(10),4(9),5,7-tetraen-13-yl]methanol	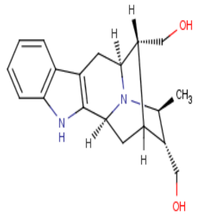
RASE0098<br><br>[4,5,23]	19(S),20(R)-dihydroperaksine-17-al	Hairy Root culture	(1S,12S,13R,15R,16S)-15-(hydroxymethyl)-16-methyl-3,17-diazapentacyclo[12.3.1.0^{2,10}.0^{4,9}.0^{12,17}]octadeca-2(10),4(9),5,7-tetraene-13-carbaldehyde	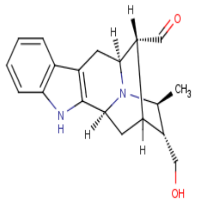
RASE0099<br><br>[4,5,23]	10-Hydroxy-19(S),20(R)-dihydroperaksine	Hairy Root culture	(1S,12S,13R,15R,16S)-13,15-bis(hydroxymethyl)-16-methyl-3,17-diazapentacyclo[12.3.1.0^{2,10}.0^{4,9}.0^{12,17}]octadeca-2(10),4(9),5,7-tetraen-7-ol	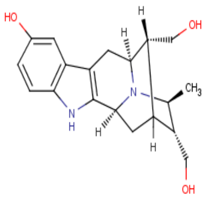

RASE0100<br><br>[23]	19(S),20(R)-(O)-Acetylpreperakine	Hairy Root culture	[(1S,12S,13R,15R,16S)-15-formyl-16-methyl-3,17-diazapentacyclo[12.3.1.0^{2,10}.0^{4,9}.0^{12,17}]octadeca-2(10),4(9),5,7-tetraen-13-yl]methyl acetate	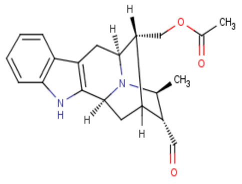
RASE0101<br><br>[11]	Reserpoxidine	Stem, Leaves, Roots, Root bark	Methyl (1R,15S,17R,18R,19S,20S)-6,18-dimethoxy-13-oxo-17-(3,4,5-trimethoxybenzoyloxy)-3,13$l^{5}-diazapentacyclo[11.8.0.0^{2,10}.0^{4,9}.0^{15,20}]henicosa-2(10),4,6,8-tetraene-19-carboxylate	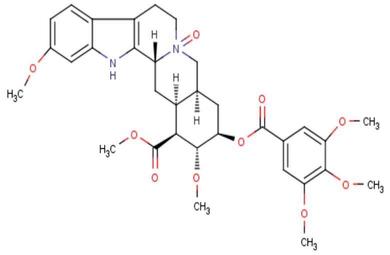
RASE0102<br><br>[4]	12-Hydroxyajmaline	Hairy Root culture	13-ethyl-8-methyl-8,15-diazahexacyclo[14.2.1.0^{1,9}.0^{2,7}.0^{10,15}.0^{12,17}]nonadeca-2(7),3,5-triene-6,14,18-triol	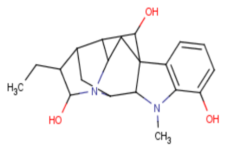
RASE0103<br><br>[4,8,11]	3-Epi-alpha-yohimbine	Hairy Root culture	Methyl (1R,15S,18S,19S,20S)-18-hydroxy-3,13-diazapentacyclo[11.8.0.0^{2,10}.0^{4,9}.0^{15,20}]henicosa-2(10),4,6,8-tetraene-19-carboxylate	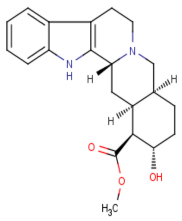

RASE0104<br><br>[4]	18-beta-hydroxy-3-epi-alpha-yohimbine	Hairy Root culture	Methyl (1S,15R,17S,18S,19R,20R)-17,18-dihydroxy-3,13-diazapentacyclo[11.8.0.0^{2,10}.0^{4,9}.0^{15,20}]henicosa-2(10),4,6,8-tetraene-19-carboxylate	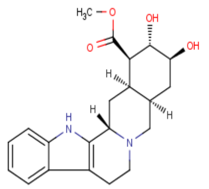
RASE0105<br><br>[4,33]	17-O-acetyl-ajmaline	Hairy Root culture, Roots	(1R,9R,10S,12R,13S,14R,16S)-18-(acetyloxy)-13-ethyl-14-hydroxy-8-methyl-8,15-diazahexacyclo[14.2.1.0^{1,9}.0^{2,7}.0^{10,15}.0^{12,17}]nonadeca-2,4,6-trien-15-ium	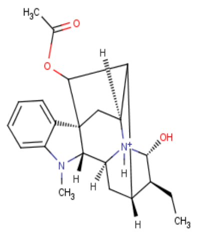
RASE0106<br><br>[4,5,18]	Strictosidine	Hairy Root culture	Methyl(2S,3R,4S)-3-ethenyl-4-[[(1S)-2,3,4,9-tetrahydro-1H-pyrido[3,4-b]indol-1-yl]methyl]-2-[(2S,3R,4S,5S,6R)-3,4,5-trihydroxy-6-(hydroxymethyl)oxan-2-yl]oxy-3,4-dihydro-2H-pyran-5-carboxylate	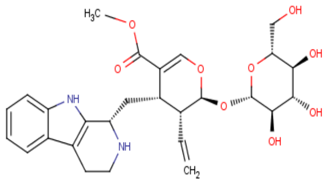
RASE0107<br><br>[4]	Strictosidine lactam	Hairy Root culture	19-ethenyl-18-{[3,4,5-trihydroxy-6-(hydroxymethyl)oxan-2-yl]oxy}-17-oxa-3,13-diazapentacyclo[11.8.0.0^{2,10}.0^{4,9}.0^{15,20}]henicosa-2(10),4(9),5,7,15-pentaen-14-one	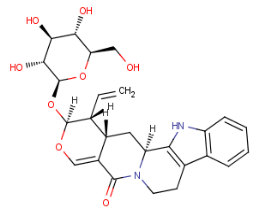

RASE0108<br><br>[7]	Eburnamonine	Cell culture	(15S,19S)-15-ethyl-1,11-diazapentacyclo[9.6.2.0^{2,7}.0^{8,18}.0^{15,19}]nonadeca-2,4,6,8(18)-tetraen-17-one	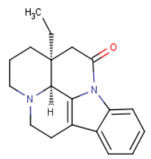
RASE0109<br><br>[7]	Stemmadenine	Cell culture	Hydroxymethyl (16Z)-16-ethylidene-4-methyl-4,14-diazatetracyclo[12.2.2.0^{3,11}.0^{5,10}]octadeca-3(11),5,7,9-tetraene-2-carboxylate	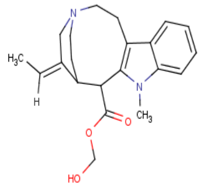
RASE0110<br><br>[7]	Akuammigine	Cell culture	Methyl (1R,15S,16S,20S)-16-methyl-17-oxa-3,13-diazapentacyclo[11.8.0.0^{2,10}.0^{4,9}.0^{15,20}]henicosa-2(10),4,6,8,18-pentaene-19-carboxylate	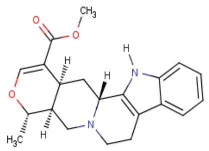
RASE0111<br><br>[7]	Gardnerine	Cell culture	[(1S,15E)-15-ethylidene-6-methoxy-3,17-diazapentacyclo[12.3.1.0^{2,10}.0^{4,9}.0^{12,17}]octadeca-2(10),4(9),5,7-tetraen-13-yl]methanol	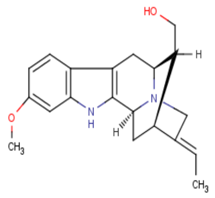

RASE0112<br><br>[7]	16-Epigardnerine	Cell culture	[(1S,12S,14S)-15-ethylidene-6-methoxy-3,17-diazapentacyclo[12.3.1.0^{2,10}.0^{4,9}.0^{12,17}]octadeca-2(10),4(9),5,7-tetraen-13-yl]methanol	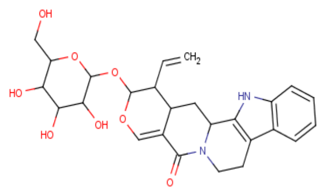
RASE0113<br><br>[7]	Isosandwicine	Roots, Cell culture	13-ethyl-8-methyl-8,15-diazahexacyclo[14.2.1.0^{1,9}.0^{2,7}.0^{10,15}.0^{12,17}]nonadeca-2(7),3,5-triene-14,18-diol	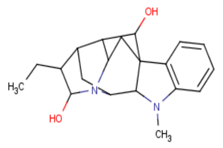
RASE0114<br><br>[7]	Rauniticine	Cell culture	Methyl (1S,15S,16R,20S)-16-methyl-17-oxa-3,13-diazapentacyclo[11.8.0.0^{2,10}.0^{4,9}.0^{15,20}]henicosa-2(10),4,6,8,18-pentaene-19-carboxylate	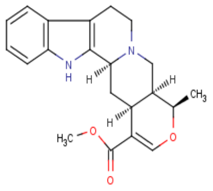
RASE0115<br><br>[7]	Sandwicine	Roots, Cell culture	(9R,14S)-13-ethyl-8-methyl-8,15-diazahexacyclo[14.2.1.0^{1,9}.0^{2,7}.0^{10,15}.0^{12,17}]nonadeca-2(7),3,5-triene-14,18-diol	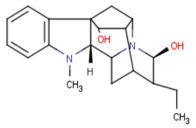

RASE0116<br><br>[7]	Vincoside lactam	Cell culture	19-ethenyl-18-{[3,4,5-trihydroxy-6-(hydroxymethyl)oxan-2-yl]oxy}-17-oxa-3,13-diazapentacyclo[11.8.0.0^{2,10}.0^{4,9}.0^{15,20}]henicosa-2(10),4(9),5,7,15-pentaen-14-one	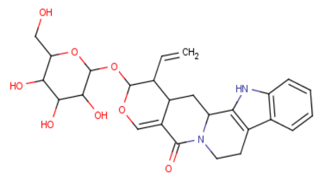
RASE0117<br><br>[7]	Alpha-yohimbine	Cell culture	Methyl (1S,15S,18S,19S,20S)-18-hydroxy-3,13-diazapentacyclo[11.8.0.0^{2,10}.0^{4,9}.0^{15,20}]henicosa-2(10),4,6,8-tetraene-19-carboxylate	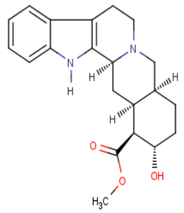
RASE0118<br><br>[7]	Beta-yohimbine	Cell culture	Methyl (1S,15R,18R,19R,20S)-18-hydroxy-3,13-diazapentacyclo[11.8.0.0^{2,10}.0^{4,9}.0^{15,20}]henicosa-2(10),4,6,8-tetraene-19-carboxylate	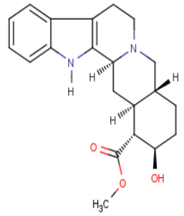
RASE0119<br><br>[7]	17,21-O-Diacetylajmaline	Cell culture	(14S)-14-(acetyloxy)-13-ethyl-8-methyl-8,15-diazahexacyclo[14.2.1.0^{1,9}.0^{2,7}.0^{10,15}.0^{12,17}]nonadeca-2(7),3,5-trien-18-yl acetate	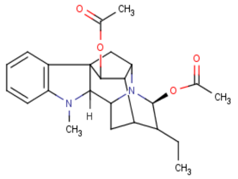

RASE0120<br><br>[7]	21-O-Acetylvomilenine	Cell Culture	(10S,13E,14S)-14-(acetyloxy)-13-ethylidene-8,15-diazahexacyclo[14.2.1.0^{1,9}.0^{2,7}.0^{10,15}.0^{12,17}]nonadeca-2(7),3,5,8-tetraen-18-yl acetate	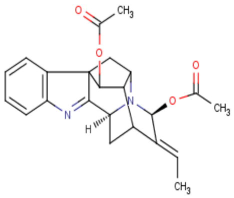
RASE0121<br><br>[22]	21-hydroxyraumacline	Cell Suspension Culture	(1S,12S,13S,14R,17S,18S)-17-ethyl-3-methyl-15-oxa-3,20-diazapentacyclo[10.7.1.0^{2,10}.0^{4,9}.0^{13,18}]icosa-2(10),4(9),5,7-tetraene-14,16-diol	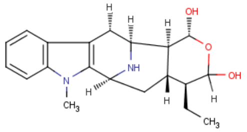
RASE0122<br><br>[34]	Tubotaiwine	Hybrid Cell Culture	Methyl 18-ethyl-8,14-diazapentacyclo[9.5.2.0^{1,9}.0^{2,7}.0^{14,17}]octadeca-2,4,6,9-tetraene-10-carboxylate	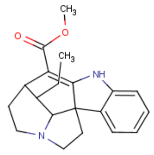
RASE0123<br><br>[34]	21-Hydroxysarpagan-glucoside	Hybrid Cell Culture	(1S,12S,14S,15E,16R)-15-ethylidene-16-{[3,4,5-trihydroxy-6-(hydroxymethyl)oxan-2-yl]oxy}-3,17-diazapentacyclo[12.3.1.0^{2,10}.0^{4,9}.0^{12,17}]octadeca-2(10),4(9),5,7-tetraene-13-carbaldehyde	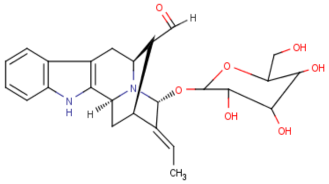

RASE0124<br><br>[34]	17-O-Acetylraugluci ne	Hybrid Cell Culture	(1R,9R,13S,14R,16S)-13-ethyl-8-methyl-14-{[3,4,5-trihydroxy-6-(hydroxymethyl)oxan-2-yl]oxy}-8,15-diazahexacyclo[14.2.1.0^{1,9}.0^{2,7}.0^{10,15}.0^{12,17}]nonadeca-2(7),3,5-trien-18-yl acetate	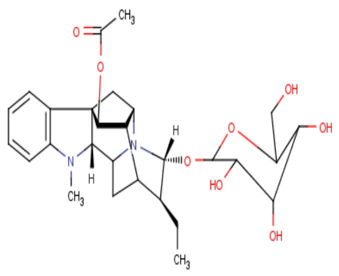
RASE0125<br><br>[34]	17-O-Acetyl-nortetraphyllici ne	Hybrid Cell Culture, Cell Suspension Culture	(1R,9R,13E,16S)-13-ethylidene-8,15-diazahexacyclo[14.2.1.0^{1,9}.0^{2,7}.0^{10,15}.0^{12,17}]nonadeca-2(7),3,5-trien-18-yl acetate	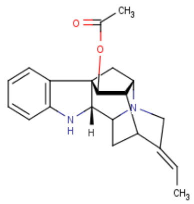
RASE0126<br><br>[34]	17-O-Acetyltetraphyl licine	Hybrid Cell Culture, Cell Suspension Culture	(1R,9R,13E,16S)-13-ethylidene-8-methyl-8,15-diazahexacyclo[14.2.1.0^{1,9}.0^{2,7}.0^{10,15}.0^{12,17}]nonadeca-2(7),3,5-trien-18-yl acetate	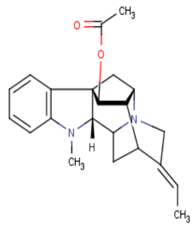
RASE0127<br><br>[34]	Suaveoline	Hybrid Cell Culture	(1S,12S)-17-ethyl-3-methyl-3,15,20-triazapentacyclo[10.7.1.0^{2,10}.0^{4,9}.0^{13,18}]icosa-2(10),4(9),5,7,13,15,17-heptaene	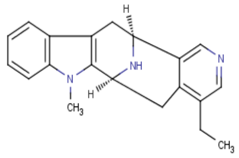

RASE0128<br><br>[34]	Macrophylline	Hybrid Cell Culture	2-{20-methyl-3,15,20-triazapentacyclo[10.7.1.0^{2,10}.0^{4,9}.0^{13,18}]icosa-2(10),4(9),5,7,13,15,17-heptaen-17-yl}ethan-1-ol	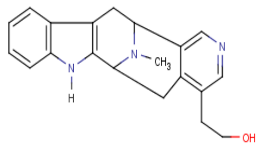
RASE0129<br><br>[34]	Rhazinilam	Hybrid Cell Culture	(12R)-12-ethyl-8,16-diazatetracyclo[10.6.1.0^{2,7}.0^{16,19}]nonadeca-1(19),2(7),3,5,17-pentaen-9-one	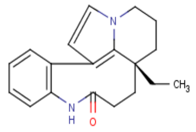
RASE0130<br><br>[34]	Acetylcorynanthine	Roots	Methyl (1S,15R,18S,19S,20S)-18-(acetyloxy)-3,13-diazapentacyclo[11.8.0.0^{2,10}.0^{4,9}.0^{15,20}]henicosa-2(10),4(9),5,7-tetraene-19-carboxylate	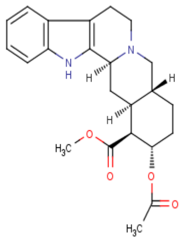
RASE0131<br><br>[34]	Raunescine	Roots	Methyl (1R,15S,17R,18R,19S,20S)-18-hydroxy-17-(3,4,5-trimethoxybenzoyloxy)-3,13-diazapentacyclo[11.8.0.0^{2,10}.0^{4,9}.0^{15,20}]henicosa-2(10),4,6,8-tetraene-19-carboxylate	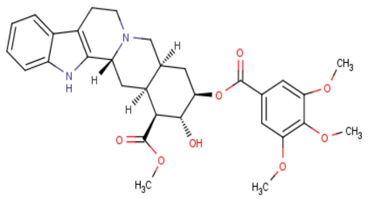

RASE0132 [27]	Isosandwicimine	Roots	(1R,9R,10S,12R,13R,14S,16S,18S)-13-ethyl-18-hydroxy-8-methyl-8,15-diazahexacyclo[14.2.1.0^{1,9}.0^{2,7}.0^{10,15}.0^{12,17}]nonadeca-2(7),3,5-trien-14-yl acetate	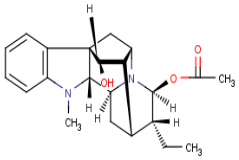
RASE0133 [27]	Seredine	Roots	Methyl 18-hydroxy-6,7-dimethoxy-3,13-diazapentacyclo[11.8.0.0^{2,10}.0^{4,9}.0^{15,20}]henicosa-2(10),4(9),5,7-tetraene-19-carboxylate	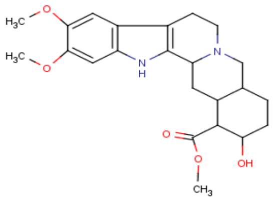
RASE0134 [8]	Ajmalicidine	Roots	Methyl (1S,15R,16S,18S,20R)-18-hydroxy-16-methyl-17-oxa-3,13-diazapentacyclo[11.8.0.0^{2,10}.0^{4,9}.0^{15,20}]henicosa-2(10),4(9),5,7-tetraene-3-carboxylate	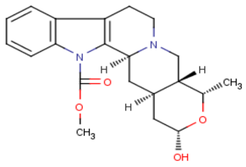
RASE0135 [8,27]	Ajmalinimine	Roots	4-acetyl-13-ethyl-14-hydroxy-8-methyl-8,15-diazahexacyclo[14.2.1.0^{1,9}.0^{2,7}.0^{10,15}.0^{12,17}]nonadeca-2,4,6-trien-18-yl acetate	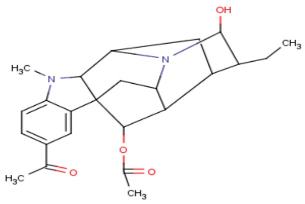

RASE0136<br><br>[8]	Raugalline	Roots	(9R,14R)-13-ethyl-8-methyl-8,15-diazahexacyclo[14.2.1.0^{1,9}.0^{2,7}.0^{10,15}.0^{12,17}]nonadeca-2,4,6-triene-14,18-diol	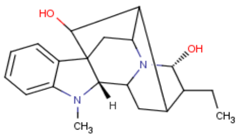
RASE0137<br><br>[8]	Perakenine	Roots	13-ethyl-8-methyl-19-oxa-8,15-diazahexacyclo[14.3.1.0^{1,9}.0^{2,7}.0^{10,15}.0^{12,17}]icosa-2(7),3,5-trien-18-ol	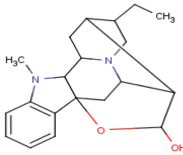
RASE0138<br><br>[8]	Serpine	Roots	Methyl 18-hydroxy-3,13-diazapentacyclo[11.8.0.0^{2,10}.0^{4,9}.0^{15,20}]henicosa-2(10),4(9),5,7-tetraene-19-carboxylate	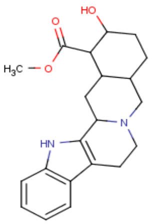
RASE0139<br><br>[8]	Acetylajmalicidine	Roots	Methyl (1S,15R,16S,18R,20R)-18-(acetyloxy)-16-methyl-17-oxa-3,13-diazapentacyclo[11.8.0.0^{2,10}.0^{4,9}.0^{15,20}]henicosa-2(10),4(9),5,7-tetraene-3-carboxylate	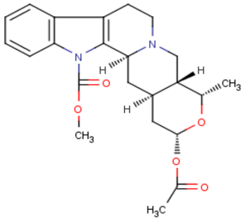

RASE0140<br><br>[8]	Acetylsandwicoline	Roots	(2S)-2-[(1S,2R,10R,12S,14R,17S)-17-(acetyloxy)-3,16-dimethyl-3,16-diazapentacyclo[10.3.1.1^{10,13}.0^{2,10}.0^{4,9}]heptadeca-4(9),5,7-trien-14-yl]-2-methylbutyl acetate	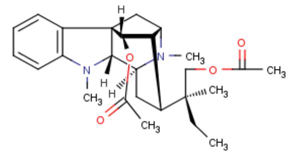
RASE0141<br><br>[8]	Acetylsandwicolidine	Roots	(9S,10S,12R,13S,14R,18S)-14-ethyl-13-methoxy-8-methyl-8,15-diazahexacyclo[14.2.1.0^{1,9}.0^{2,7}.0^{10,15}.0^{12,17}]nonadeca-2(7),3,5-trien-18-yl acetate	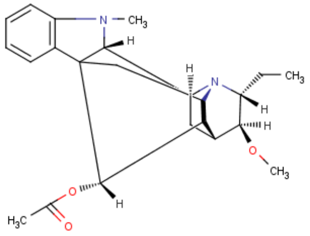
RASE0142<br><br>[5,35]	Dehydrogeissoschizine	Cell Suspension Culture	(2S,4S,5E)-5-ethylidene-4-(1-methoxy-1,3-dioxopropan-2-yl)-7$l^{5},17-diazatetracyclo[8.7.0.0^{2,7}.0^{11,16}]heptadeca-1(10),6,11,13,15-pentaen-7-ylium	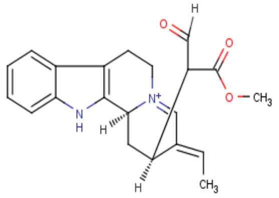

RASE0143<br><br>[5]	19(S),20(R)-dihydroperaksine-17,21-al	Cell Suspension Culture	(1S,12S,15R,16S)-16-methyl-3,17-diazapentacyclo[12.3.1.0^{2,10}.0^{4,9}.0^{12,17}]octadeca-2(10),4,6,8-tetraene-13,15-dicarbaldehyde	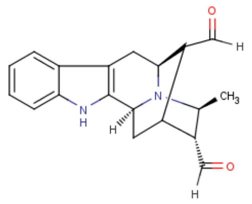
RASE0144<br><br>[33]	(+)-17R-O-(3',4',5'-trimethoxybenzoyl)ajmaline	Roots	(13S,14R)-13-ethyl-14-hydroxy-8-methyl-8,15-diazahexacyclo[14.2.1.0^{1,9}.0^{2,7}.0^{10,15}.0^{12,17}]nonadeca-2(7),3,5-trien-18-yl 3,4,5-trimethoxybenzoate	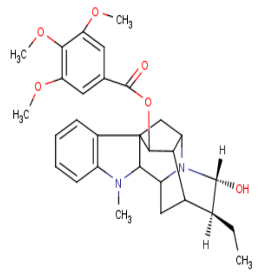
RASE0145<br><br>[21]	4,21-secoajmaline	Cell Culture	14-[(2S)-1-hydroxybutan-2-yl]-3-methyl-3,16-diazapentacyclo[10.3.1.1^{10,13}.0^{2,10}.0^{4,9}]heptadeca-4(9),5,7-trien-17-ol	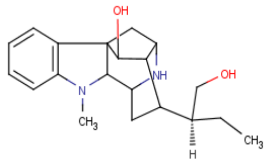

RASE0146<br><br>[27]	Ajmalicimine	Roots	Methyl (4S,8S,9R)-8-methyl-7-oxa-11,21-diazatetracyclo[12.7.0.0^{4,9}.0^{15,20}]henicosa-1(14),5,15(20),16,18-pentaene-5-carboxylate	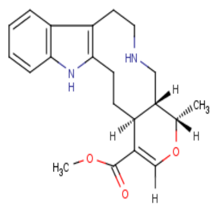
RASE0147<br><br>[27]	Acetylajmalicimine	Roots	Methyl (4S,8S,9R)-11-acetyl-8-methyl-7-oxa-11,21-diazatetracyclo[12.7.0.0^{4,9}.0^{15,20}]henicosa-1(14),5,15(20),16,18-pentaene-5-carboxylate	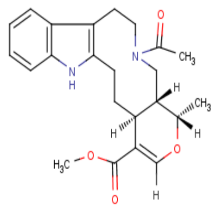

REFERENCES

1. Madhusudanan K, Banerjee S, Khanuja S, Chattopadhyay S (2008) Analysis of hairy root culture of Rauvolfia serpentina using direct analysis in real time mass spectrometric technique. Biomedical Chromatography 22: 596–600. doi:10.1002/bmc.974.

2. Srivastava A, Tripathi A, Pandey R, Verma R, Gupta M (2006) Quantitative determination of reserpine, ajmaline, and ajmalicine in Rauvolfia serpentina by reversed-phase high-performance liquid chromatography. Journal of Chromatographic Science 44: 557–560. doi:10.1093/chromsci/44.9.557.

3. Varchi G, Battaglia A, Samorì C, Baldelli E, Danieli B, et al. (2005) Synthesis of Deserpidine from Reserpine. Journal of Natural Products 68: 1629–1631. doi:10.1021/np050179x.

4. Sheludko Y, Gerasimenko I, Kolshorn H, Stöckigt J (2002) New alkaloids of the sarpagine group from Rauvolfia serpentina hairy root culture. Journal of Natural Products 65: 1006–1010. doi:10.1021/np0200919.

5. Sun L, Ruppert M, Sheludko Y, Warzecha H, Zhao Y, et al. (2008) Purification, cloning, functional expression and characterization of perakine reductase: the first example from the AKR enzyme family, extending the alkaloidal network of the plant Rauvolfia. Plant Molecular Biology 67: 455–467. doi:10.1007/s11103-008-9331-7.

6. Yarnell E (2007) Rauvolfia serpentinia Benth ex Kurz (rauwolfia), Apocynaceae and related species.

7. Gerasimenko I, Sheludko Y, Unger M, Stöckigt J (2001) Development of an efficient system for the separation of indole alkaloids by high performance liquid chromatography and its applications. Phytochemical Analysis 12: 96–103. doi:10.1002/pca.567.

8. Zaidi SSA (1986) Studies in the alkaloidal constituents of the roots of rauwolfia serpentina benth with particular regional variations and synthetic extensions of von braun (BrCN) reaction on alkaloidal and simpler organic bases. University of Karachi.

9. Wachsmuth O, Matusch R (2002) Anhydronium bases from Rauvolfia serpentina. Phytochemistry 61: 705–709. doi:10.1016/S0031-9422(02)00372-2.

10. Dassonneville L, Bonjean K, Colson P, Houssier C (1999) Stimulation of Topoisomerase II-Mediated DNA Cleavage by Three DNA-Intercalating Plant Alkaloids: Cryptolepine, Matadine, and Serpentine. Biochemistry 38: 7719–7726. doi:10.1021/bi990094t.

11. Mittal B, Meenakshi, Sharma A, Kumar V (2012) Phytochemical & Pharmacological Activity Of Rauwolfia Serpentina – A Review. International Journal Of Ayurvedic And Herbal Medicine 2: 427–434.

12. Itoh A, Kumashiro T, Yamaguchi M, Nagakura N, Mizushina Y, et al. (2005) Indole alkaloids and other constituents of Rauwolfia serpentina. Journal of Natural Products 68: 848–852. doi:10.1021/np058007n.

13. Dey A, De J (2011) Ethnobotanical aspects of Rauvolfia serpentina (L). Benth. ex Kurz. in India, Nepal and Bangladesh. Journal of Medicinal Plants Research 5: 144–150.

14. Phillips K, Horst R, Koszewski N, Simon R (2012) Vitamin d(4) in mushrooms. PloS One 7: 1–10. doi:10.1371/journal.pone.0040702.

15. India Herbs - ancient remedies for modern times. (2012). Available:http://www.cardiofy.com/index.htm.

16. GLOBinMeD-Global Information Hub On Integrated Medicine. (2012). Available:http://www.globinmed.com/index.php?option=com_content&view=article&id=79110:rauvolfia-serpentina-benth&catid=720:r#6.

17. A database on antidiabetic plants. (2012). Available:http://herbalmedicinalplants.org/index.php.

18. Ruppert M, Panjikar S, Barleben L, Stöckigt J (2006) Heterologous expression, purification, crystallization and preliminary X-ray analysis of raucaffricine glucosidase, a plant enzyme specifically involved in Rauvolfia alkaloid biosynthesis. Acta Crystallographica Section F Structural Biology and Crystallization Communications 62: 257–260. doi:10.1107/S174430910600457X.

19. Warzecha H, Obitz P, Sto J (1999) Purification, partial amino acid sequence and structure of the product of raucaffricine-O-β-D-glucosidase from plant cell cultures of Rauwolfia serpentina. Phytochemistry 50: 1099–1109. doi:10.1016/S0031-9422(98)00689-X.

20. Sheludko Y, Gerasimenko I, Kolshorn H, Stöckigt J (2002) Isolation and structure elucidation of a new indole alkaloid from Rauvolfia serpentina hairy root culture: the first naturally occurring alkaloid of the raumacline group. Planta Medica 68: 435–439. doi:10.1055/s-2002-32074.

21. Bailey PD, Beard M a., Dang HPT, Phillips TR, Price R a., et al. (2008) Debenzylation using catalytic hydrogenolysis in trifluoroethanol, and the total synthesis of (−)-raumacline. Tetrahedron Letters 49: 2150–2153. doi:10.1016/j.tetlet.2008.01.104.

22. Obitz P, Endreb S, Joachim S (1995) Enzymatic biosynthesis of raumacline. Phytochemistry 40: 1407–1417. doi:10.1016/0031-9422(95)00503-Y.

23. Edwankar R, Edwankar C, Deschamps J, Cook J (2011) Regiospecific, enantiospecific total synthesis of C-19 methyl substituted sarpagine alkaloids dihydroperaksine-17-al and dihydroperaksine. Organic Letters 13: 5216–5219. doi:10.1021/ol202101p.

24. Mattern-Dogru E, Ma X, Hartmann J, Decker H, Stöckigt J (2002) Potential active-site residues in polyneuridine aldehyde esterase, a central enzyme of indole alkaloid biosynthesis, by modelling and site-directed mutagenesis. European Journal of Biochemistry 269: 2889–2896. doi:10.1046/j.1432-1033.2002.02956.x.

25. Bayer A, Ma X, Stöckigt J (2004) Acetyltransfer in natural product biosynthesis--functional cloning and molecular analysis of vinorine synthase. Bioorganic & Medicinal Chemistry 12: 2787–2795. doi:10.1016/j.bmc.2004.02.029.

26. Ganugapati J, Baldwa A, Lalani S (2012) Docking Studies of Rauwolfia Serpentina Alkaloids as Insulin Receptor Activators. International Journal of Computer Applications 43: 32–37. doi:10.5120/6173-8600.

27. Haider SI (1986) Extended studies in the alkaloidal constituents of Rauwolfia serpentina from thailland and synthetic extensions of Von braun (BrCN) reaction on alkaloidal and simpler organic bases University of Karachi.

28. Reddy MP, HariChandana P, Reddy KHV (n.d.) Isolation and characterization of Indole alkaloids from Rauwolfia serpentine. Quality Standards of Indian Medicinal Plants.

29. Vakil R (1949) A Clinical trial of Rauwolfia serpentina in essential hypertension. British Heart Journal 11: 350–355. doi:10.1136/hrt.11.4.350.

30. Ghosh B, Basu R (1955) A sterol (serposterol) for use in mental cases from Rauwolfia Serpentina. Naturwissenschaften 42: 130. doi:10.1007/BF00589414.

31. Arend J, Warzecha H, Stöckigt J (2000) Hydroquinone: O-glucosyltransferase from cultivated Rauvolfia cells: enrichment and partial amino acid sequences. Phytochemistry 53: 187–193. doi:10.1016/S0031-9422(99)00539-7.

32. Arend J, Warzecha H, Hefner T, Stöckigt J (2001) Utilizing genetically engineered bacteria to produce plant-specific glucosides. Biotechnology and Bioengineering 76: 126–131. doi:10.1002/bit.1152.

33. Hanhinen P, Lounasmaa M (2001) Revision of the structure of ajmalimine. Journal of Natural Products 64: 686–687. doi:10.1021/np000618m.

34. Stöckigt J, Sheludk Y, Unger M, Gerasimenko I, Warzecha H, et al. (2002) High-performance liquid chromatographic, capillary electrophoretic and capillary electrophoretic-electrospray ionisation mass spectrometric analysis of selected alkaloid groups. Journal of Chromatography A 967: 85–113. doi:10.1016/S0021-9673(02)00037-7.

35. Dogru E, Warzecha H, Seibel F, Haebel S, Lottspeich F, et al. (2000) The gene encoding polyneuridine aldehyde esterase of monoterpenoid indole alkaloid biosynthesis in plants is an ortholog of the a / b hydrolase super family. European Journal of Biochemistry 267: 1397–1406. doi:10.1046/j.1432-1327.2000.01136.x.
