# Supplementary material for: Prospecting for Novel Plant-Derived Molecules of Rauvolfia serpentina as Inhibitors of Aldose Reductase, a Potent Drug Target for Diabetes and Its Complications
Source: PLoS One. 2013 Apr 17;8(4):e61327. doi: 10.1371/journal.pone.0061327 (PMC3629236; doi:10.1371/journal.pone.0061327)
Supplement: Table S2 — Nine R. serpentina PDMs with desired binding affinities. (PDF) [file pone.0061327.s004.pdf]

Table S2. Nine *R. serpentina* PDMs with desirable binding affinities. Results of docking runs for the nine PDMs with binding affinity consistently better than the positive control (-10.7 kcal/mol), against Aldose Reductase (PDB ID: 1US0).

**Ligand**	**PDM Name**	**Binding affinity(kcal/mol)[number of modes]**				
		**Run 1**	**Run 2**	**Run 3**	**Run 4**	**Run 5**
RASE0007	Yohimbine	-11.1 [03]	--	-11.1 [01]	--	--
RASE0048	Indobine	-11.0 [12]	-11.0 [20]	-11.0 [20]	-11.1 [19]	-11.0 [14]
RASE0049	Indobinine	-11.1 [14]	-11.0 [18]	-11.0 [10]	-11.0 [12]	-11.0 [14]
RASE0070	Yohimbinic acid	--	-11.6[01]	-11.6[01]	--	-11.6[02]
RASE0071	Isorauhimbinic acid	--	--	-11.6[02]	--	--
RASE0125	17-O-Acetyl-nortetraphyllicine	-10.7[04]	-10.7[04]	-10.7[04]	-10.6[05]	-10.7[04]
RASE0126	17-O-Acetyltetraphyllicine	-10.9[03]	-10.9[04]	-10.9[03]	-10.9[03]	-10.9[03]
RASE0142	Dehydrogeissoschizine	-11.1 [01]	-11.1 [01]	-11.1 [01]	-11.1 [01]	-11.1 [01]
RASE0143	19(S),20(R)-dihydroperaksine-17,21-al	-10.7[03]	-10.7[03]	-10.7[04]	-10.7[02]	-10.7[04]
