# Supplementary material for: Prospecting for Novel Plant-Derived Molecules of Rauvolfia serpentina as Inhibitors of Aldose Reductase, a Potent Drug Target for Diabetes and Its Complications
Source: PLoS One. 2013 Apr 17;8(4):e61327. doi: 10.1371/journal.pone.0061327 (PMC3629236; doi:10.1371/journal.pone.0061327)
Supplement: Table S3 — Hydrogen bonding pattern with critical residues (for best PDMs). (PDF) [file pone.0061327.s005.pdf]

Table S3. Hydrogen bonding pattern with critical residues (for best PDMs). Details of critical interactions during MD simulations at every 0.5ns.

**Best PDMs**	**0.5ns**	**1.0ns**	**1.5ns**	**2.0ns**	**2.5ns**	**3.0ns**	**3.5ns**	**4.0ns**	**4.5ns**	**5.0ns**
RASE0048	His110<br>Trp111	His110<br>Trp111	His110<br>Trp111	His110<br>Trp111	His110<br>Trp111	His110<br>Trp111	Cys298	His110<br>Trp111	His110<br>Trp111	His110<br>Trp111
RASE0049	Trp20	His110	--	--	His110<br>Trp20	His110<br>Trp111	His110	His110	His110<br>Trp111	His110<br>Trp111
RASE0143	His110	His110<br>Trp111	His110<br>Gln49	Trp20	--	--	--	--	--	Gln49
