# Supplementary material for: Prospecting for Novel Plant-Derived Molecules of Rauvolfia serpentina as Inhibitors of Aldose Reductase, a Potent Drug Target for Diabetes and Its Complications
Source: PLoS One. 2013 Apr 17;8(4):e61327. doi: 10.1371/journal.pone.0061327 (PMC3629236; doi:10.1371/journal.pone.0061327)
Supplement: Table S4 — Binding affinities and hydrogen bond interactions for analogs of RASE0048. (PDF) [file pone.0061327.s006.pdf]

Table S4. Binding affinities and hydrogen bond interactions for analogs of RASE0048. Results of docking runs for ZINC analogs of indobine (RASE0048) with binding affinity consistently better as compared that of the positive control (-10.7 kcal/mol), against Aldose Reductase (PDB ID: 1US0). For few of the analogs, there were multiple modes/poses with desirable binding affinity. Results of docking runs of indobine are mentioned for comparison (highlighted). Among the analogs of RASE0048, data for the representative molecule chosen for MD analysis (ZINC04286771) is shown in bold.

PDM_ID	Mode No.	Binding affinity(kcal/mol)[number of modes]					Hydrogen interactions[bond length]				
		Run 1	Run 2	Run 3	Run 4	Run 5	Run 1	Run 2	Run 3	Run 4	Run 5
RASE0048	1	-11.0[12]	-11.0[20]	-11.0[20]	-11.1[19]	-11.0[14]	Trp111[3.27]<br>His110 [2.94]	Trp111[3.23]<br>His110[3.02]	Trp111[3.07]<br>His110[3.08]	Trp111[3.14]<br>His110[3.11]	Trp111[3.08]<br>His110[3.00]
ZINC08650120	1	-11.2[14]	-11.0[19]	-11.1[16]	-11.0[20]	-11.0[20]	Trp111[3.20]<br>His110 [2.96]	Trp111[3.05]<br>His110[3.08]	Trp111[3.16]<br>His110[3.00]	Trp111[3.11]<br>His110[3.10]	His110[3.06]
ZINC04286938	1	-11.6[20]	-11.6[16]	-11.6[20]	-11.4[20]	-11.6[16]	Trp111[2.94]	Trp111[3.05]<br>His110[3.12]	Trp111[2.98]	Nil	Trp111[3.22]<br>His110[3.03]
ZINC04286938	2	-10.8[20]	--	-10.9[20]	-11.2[20]	-11.1[16]	Trp111[3.30]<br>His110[3.15]<br>Tyr48[3.21]<br>Asp43[3.18]<br>Gln183[2.94]	--	Nil	Trp111[3.00]	Nil
ZINC04286938	3	--	--	-10.7[20]	-10.7[20]	-10.7[16]	--	--	Trp111[3.25]<br>His110[3.27]<br>Tyr48[3.27]<br>Asp43[3.17]<br>Gln183[3.02]	Nil	Nil
ZINC04286938	4	--	--	--	--	-10.7[16]	--	--	--	--	Trp111[3.22]<br>His110[3.15]<br>Tyr48[3.26]<br>Asp43[3.18]<br>Gln183[2.97]
ZINC08650118	1	-11.3[20]	-11.4[20]	-11.4[19]	-11.4[20]	-11.4[18]	His110[3.06]	Trp111[3.22]<br>His110[2.98]	Trp111[3.23]<br>His110[3.00]	Trp111[3.26]<br>His110[2.97]	Trp111[3.35]<br>His110[3.02]
ZINC08650118	2	-10.7[20]	-10.7[20]	-10.8[19]	-10.8[20]	-10.9[18]	His110[3.21]<br>Tyr48[2.80]	Trp111[2.98]<br>His110[3.03]	Trp111[3.07]<br>Tyr48[2.98]	His110[3.31]<br>Tyr48[2.95]	Trp111[3.13]<br>Tyr48[3.05]

								Tyr48[3.25]<br>Asp43[3.15]<br>Gln183[3.06]	Gln183[2.72]		Gln183[2.81]<br>Thr113[3.15]
ZINC08650118	3	--	--	-10.7[19]	-10.7[20]	-10.8[18]	--	--	Trp111[3.24]<br>His110[3.01]<br>Val47[3.34]<br>Tyr48[3.26]<br>Asp43[3.15]<br>Gln183[2.97]	Trp111[3.28]<br>His110[2.99]<br>Tyr48[3.26]<br>Asp43[3.18]<br>Gln183[2.95]	Trp111[3.24]<br>His110[3.05]<br>Tyr48[3.27]<br>Asp43[3.15]<br>Gln183[3.02]
**ZINC04286771**	**1**	**-11.4[20]**	**-11.4[20]**	**-11.4[20]**	**-11.3[19]**	**-11.4[16]**	**Trp111[3.07]<br>His110[3.00]**	**Trp111[3.01]<br>His110[2.99]**	**Trp111[3.11]<br>His110[2.97]**	**Trp111[3.10]<br>His110[2.95]**	**Trp111[3.16]<br>His110[2.99]**
ZINC04286771	2	-10.7[20]	-10.7[20]	-10.7[20]	--	-10.7[16]	Nil	Nil	Nil	--	Nil
ZINC08650121	1	-11.1[20]	-11.0[19]	-11.1[20]	-10.9[20]	-10.9[19]	Trp111[2.96]<br>His110[2.96]	Trp111[3.23]<br>His110[3.00]	Trp111[2.89]<br>His110[2.93]	Trp111[3.22]<br>His110[3.03]	Trp111[3.28]<br>His110[3.01]
ZINC08650121	2	--	--	-10.7[20]	--	--	--	--	Nil	--	--
ZINC04286761	1	-11.1[20]	-11.0[19]	-11.0[20]	-11.2[20]	-11.1[19]	Trp111[3.29]<br>His110[3.03]	Trp111[3.29]<br>His110[3.05]	Trp111[3.31]<br>His110[3.01]	Trp111[2.94]<br>His110[3.02]	Trp111[3.31]<br>His110[3.01]
ZINC04286761	2	--	-11.0[19]	--	--	--	--	Trp111[3.14]<br>His110[3.03]	--	--	--
ZINC03457470	1	-11.3[20]	-11.1[20]	-11.3[20]	-11.2[20]	-11.3[20]	Trp111[3.12]<br>His110[2.95]	Trp111[3.21]<br>His110[3.03]	Trp111[3.18]<br>His110[3.00]	Trp111[3.03]<br>His110[3.05]	Trp111[3.22]<br>His110[2.98]
ZINC03457470	2	-10.7[20]	-10.7[20]	-10.7[20]	-11.1[20]	-10.7[20]	Nil	Trp111[3.17]<br>His110[3.10]<br>Tyr48[3.27]<br>Asp43[3.22]<br>Gln183[3.05]	Trp111[3.18]<br>His110[3.11]<br>Tyr48[3.22]<br>Asp43[3.17]<br>Gln183[2.97]	Trp111[3.23]<br>His110[2.98]	Trp111[3.16]<br>His110[3.13]<br>Tyr48[3.25]<br>Asp43[3.19]<br>Gln183[3.00]
ZINC03457470	3	-10.7[20]	--	--	-10.8[20]	--	Trp111[3.19]<br>His110[3.10]<br>Tyr48[3.24]<br>Asp43[3.17]<br>Gln183[2.98]	--	--	Trp111[3.17]<br>His110[3.11]<br>Tyr48[3.23]<br>Asp43[3.16]<br>Gln183[2.95]	--
ZINC04286496	1	-11.9[19]	-11.7[15]	-11.3[20]	-11.7[19]	-11.7[20]	Trp111[3.12]<br>His110[3.10]	Ser210[3.33]	Ser210[3.23]	Trp111[2.95]<br>His110[3.10]	Trp111[3.17]

							Ser210[3.18]			Ser210[3.16]	
ZINC04286496	2	-10.9[19]	-11.2[15]	-11.1[20]	-11.5[19]	-10.9[20]	Thr113[3.09]<br>Trp111[3.01]<br>Tyr48[3.07]<br>Gln183[2.76]	Trp111[3.12]<br>Ser159[2.44]	Trp111[3.18]<br>Ser159[2.44]	Nil	Ser159[2.39]
ZINC04286496	3	-10.9[19]	-10.8[15]	--	-11.1[19]	-10.8[20]	Nil	His110[2.89]<br>Ser210[3.28]	--	Trp111[2.99]<br>Ser159[2.36]	Nil
ZINC04286496	4	-10.8[19]	-10.7[15]	--	--	-10.8[20]	Trp111[3.24]<br>His110[3.25]<br>Tyr48[3.23]<br>Asp43[3.15]<br>Gln183[2.99]	His110[3.10]<br>Tyr48[3.19]<br>Asp43[3.19]<br>Gln183[2.98]	--	--	His110[3.20, 3.20]<br>Tyr48[3.24]<br>Asp43[3.19]<br>Gln183[3.03]
ZINC04286496	5	-10.8[19]	--	--	--	--	Ser210[3.20]<br>Ser214[3.20]	--	--	--	--
ZINC03273473	1	-11.3[19]	-11.3[15]	-11.3[17]	-11.0[17]	-11.3[15]	Trp111[2.99]<br>His110[3.02]	Trp111[3.26]<br>His110[3.02]	Trp111[3.10]<br>His110[3.08]	Nil	Nil
ZINC03273473	2	-11.1[19]	-11.1[15]	--	-10.9[17]	--	Nil	Trp111[2.81]	--	Nil	Trp111[3.25]<br>His110[3.12]<br>Tyr48[3.22]<br>Asp43[3.18]<br>Gln183[2.95]
ZINC03273473	3	-10.8[19]	--	--	--	--	His110[3.20]	--	--	--	--
ZINC08649692	1	-11.3[20]	-11.3[17]	-11.4[17]	-11.4[20]	-11.3[19]	Trp111[3.08]<br>His110[3.11]	Trp111[3.03]<br>His110[3.16]	Trp111[3.08]<br>His110[3.15]	Trp111[3.22]<br>His110[3.12]	Trp111[3.32]<br>His110[3.08]
ZINC08649692	2	-11.2[20]	--	--	-11.3[20]	-10.7[19]	Trp111[3.17]<br>His110[3.02]	Nil	Nil	Trp111[3.17]<br>His110[2.95]	
