# Supplementary material for: Prospecting for Novel Plant-Derived Molecules of Rauvolfia serpentina as Inhibitors of Aldose Reductase, a Potent Drug Target for Diabetes and Its Complications
Source: PLoS One. 2013 Apr 17;8(4):e61327. doi: 10.1371/journal.pone.0061327 (PMC3629236; doi:10.1371/journal.pone.0061327)
Supplement: Table S5 — Binding affinities and hydrogen bond interactions for analogs of RASE0049. (PDF) [file pone.0061327.s007.pdf]

Table S5. Binding affinities and hydrogen bond interactions for analogs of RASE0049. Results of docking runs for ZINC analogs of indobinine (RASE0049) with binding affinities consistently better than that of the positive control (-10.7 kcal/mol), against Aldose Reductase (PDB ID: 1US0). Results of docking runs of indobinine are mentioned for comparison (highlighted). Among the analogs of RASE0049, data for the representative molecule chosen for MD analysis (ZINC49016166) is shown in bold.

PDM_ID	Mode No.	Binding affinity(kcal/mol)[number of modes]					Hydrogen bond interactions[bond length]				
		Run 1	Run 2	Run 3	Run 4	Run 5	Run 1	Run 2	Run 3	Run 4	Run 5
RASE0049	1	-11.1[14]	-11.0[18]	-11.0[10]	-11.0[12]	-11.0[14]	Trp111[3.20]	Trp111[3.21]	Trp111[3.12]	Trp111[3.12]	Trp111[3.09]
ZINC14517206	1	-11.1[13]	-11.1[13]	-11.1[13]	-11.1[15]	-11.1[13]	Trp111[3.14]	Trp111[3.18]	Trp111[3.14]	Trp111[3.17]	Trp111[3.09]
ZINC66777285	1	-10.7[15]	-10.8[17]	--	--	--	Nil	Nil	--	--	--
ZINC66777292	1	-10.7[18]	--	-10.7[18]	--	-10.7[17]	Trp111[3.07]	Asn160[2.89]	Asn160[2.89]	Asn160[2.89]	Trp111[3.01] His110[3.03]
**ZINC49016166**	**1**	**-11.0[09]**	**-11.1[10]**	**-11.1[11]**	**-11.1[16]**	**-11.1[13]**	**Trp111[3.00] His110[2.91]**	**Trp111[2.99] His110[2.90]**	**Trp111[2.98] His110[2.90]**	**Trp111[2.99] His110[2.88]**	**Trp111[2.98] His110[2.91]**
ZINC04286775	1	-11.3[13]	-11.4[11]	-11.3[12]	-11.3[10]	-11.3[14]	Trp111[3.12] Asn160[2.91]	Trp111[3.18] Asn160[2.93]	Trp111[3.18] Asn160[2.91]	Trp111[3.12] His110[3.05] Asn160[2.89]	Trp111[3.03] Asn160[2.90]
ZINC04286774	1	-10.9[15]	-11.0[19]	-11.0[17]	-11.0[13]	-11.0[18]	Trp111[2.92]	Trp111[2.93]	Trp111[2.92]	Trp111[2.92]	Trp111[2.93]
ZINC57967803	1	-10.8[20]	-10.8[15]	-10.8[15]	-10.8[14]	-10.7[18]	His110[2.81]	His110[2.86]	His110[3.05]	His110[2.83]	His110[2.80]
ZINC08650109	1	--	--	-10.7[20]	--	--	--	--	Nil	--	--
