# Supplementary material for: Prospecting for Novel Plant-Derived Molecules of Rauvolfia serpentina as Inhibitors of Aldose Reductase, a Potent Drug Target for Diabetes and Its Complications
Source: PLoS One. 2013 Apr 17;8(4):e61327. doi: 10.1371/journal.pone.0061327 (PMC3629236; doi:10.1371/journal.pone.0061327)
Supplement: Table S6 — Sixteen indole alkaloids obtained by screening the structural analogs of two PDM leads. (PDF) [file pone.0061327.s008.pdf]

Table S6. Sixteen indole alkaloids obtained by screening structural analogs of two PDM leads. A total of 16 ZINC leads were identified as potential ARIs by ligand-based screening of 22 structural analogs of the PDM leads (indobine and indobinine).

ZINC _ID	IUPAC	STRUCTURE
**Leads obtained from the analogs of indobine (RASE0048)**		
ZINC08650120	benzyl 3-(1H-indol-3-yl)propanoate	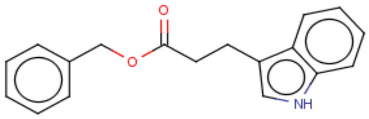
ZINC04286938	(3-methylphenyl)methyl 3-(1H-indol-3-yl)propanoate	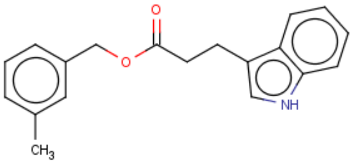
ZINC08650118	(4-cyanophenyl)methyl 3-(1H-indol-3-yl)propanoate	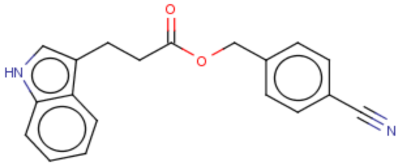
ZINC04286771	(4-fluorophenyl)methyl 3-(1H-indol-3-yl)propanoate	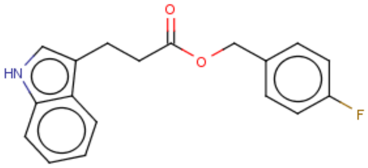
ZINC08650121	(4-bromophenyl)methyl 3-(1H-indol-3-yl)propanoate	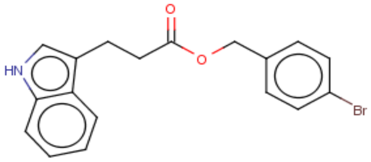
ZINC04286761	(4-chlorophenyl)methyl 3-(1H-indol-3-yl)propanoate	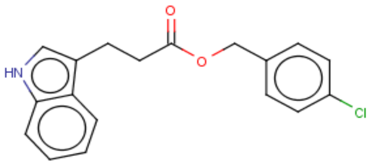
ZINC03457470	(2-fluorophenyl)methyl 3-(1H-indol-3-yl)propanoate	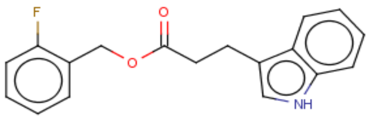
ZINC04286496	(3-cyanophenyl)methyl 3-(1H-indol-3-yl)propanoate	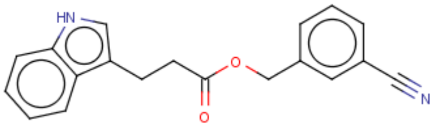

ZINC03273473	(3-chlorophenyl)methyl 3-(1H-indol-3-yl)propanoate	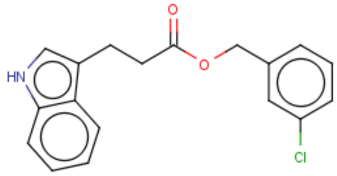
ZINC08649692	(3-fluorophenyl)methyl 3-(1H-indol-3-yl)propanoate	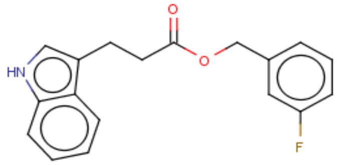
**Leads obtained from the analogs of indobinine (RASE0049)**		
ZINC14517206	cyclohexyl 3-(1H-indol-3-yl)propanoate	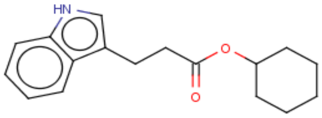
ZINC66777292	(1R,3S)-3-methoxycyclohexyl 3-(1H-indol-3-yl)propanoate	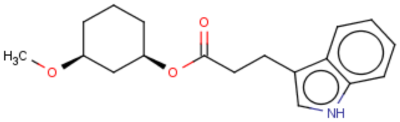
ZINC49016166	cyclopentyl 3-(1H-indol-3-yl)propanoate	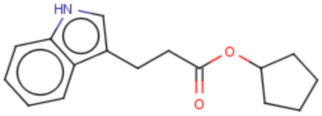
ZINC04286775	(1S)-2-oxocyclohexyl 3-(1H-indol-3-yl)propanoate	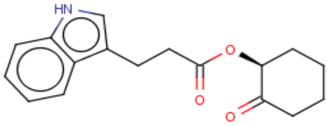
ZINC04286774	(1R)-2-oxocyclohexyl 3-(1H-indol-3-yl)propanoate	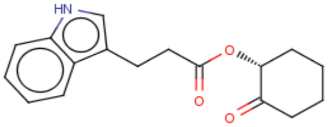
ZINC57967803	(2S)-oxan-2-ylmethyl 3-(1H-indol-3-yl)propanoate	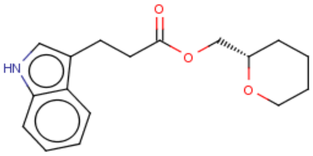
