# Supplementary material for: Prospecting for Novel Plant-Derived Molecules of Rauvolfia serpentina as Inhibitors of Aldose Reductase, a Potent Drug Target for Diabetes and Its Complications
Source: PLoS One. 2013 Apr 17;8(4):e61327. doi: 10.1371/journal.pone.0061327 (PMC3629236; doi:10.1371/journal.pone.0061327)
Supplement: Table S7 — Hydrogen bonding pattern with critical residues (for representative ZINC leads). (PDF) [file pone.0061327.s009.pdf]

Table S7. Hydrogen bonding pattern with critical residues (for representative ZINC leads). Details of critical interactions during MD simulations at every 0.5ns.

ZINC Leads	0.5ns	1.0ns	1.5ns	2.0ns	2.5ns	3.0ns	3.5ns	4.0ns	4.5ns	5.0ns
ZINC04286771	Ser302<br>His110	--	His110	--	His110	His110	His110	His110	His110	His110
ZINC49016166	His110<br>Cys298	His110	--	His110<br>Cys298	His110	His110	His110	His110	His110	His110
